# CDK4/6-targeted therapy for gastrointestinal cancers: from resistance mechanisms to immuno-combination strategies guided by biomarkers

**DOI:** 10.3389/fimmu.2026.1845921

**Published:** 2026-06-04

**Authors:** Junhao Cui, Yanchuan Zhang, Yongfang Wu, Guangjie Zhang, Jie Lan, Yingshuang Wang

**Affiliations:** 1 Sichuan Key Laboratory of Medical Molecular Testing, College of Medical Technology, Chengdu University of Traditional Chinese Medicine, Chengdu, China; 2 Department of Clinical Laboratory, The Fifth People’s Hospital Affiliated to Chengdu University of Traditional Chinese Medicine, Chengdu, China; 3 Division of Head & Neck Tumor Multimodality Treatment, Cancer Center, Institute of Breast Health Medicine, West China Hospital, Sichuan University, Chengdu, Sichuan, China

**Keywords:** CDK4/6 inhibitors, cell cycle regulation, combination therapy, drug resistance, gastrointestinal cancers, tumor immune microenvironment

## Abstract

Gastrointestinal (GI) cancers, encompassing esophageal, gastric, colorectal, hepatocellular, and pancreatic cancers, represent a major global health burden with persistently high morbidity and mortality. Given the limited therapeutic options and poor prognoses for patients with advanced-stage disease, there is an urgent and unmet clinical need for novel targeted therapies. Cyclin-dependent kinase 4/6 (CDK4/6) inhibitors (such as palbociclib, ribociclib, and abemaciclib) exert their effects by arresting the cell cycle at the G1/S checkpoint. Their landmark clinical success in hormone receptor-positive breast cancer has highlighted the therapeutic potential of targeting cell cycle regulation, thereby prompting extensive investigation into their application in solid tumors of the digestive system. Emerging evidence indicates that, beyond their direct antiproliferative effects, CDK4/6 inhibitors profoundly remodel the tumor immune microenvironment (TIME). By enhancing tumor antigen presentation, diminishing the immunosuppressive activity of regulatory T cells (Tregs), and promoting effector T cell infiltration, these agents provide a robust mechanistic rationale for synergistic combinations with immune checkpoint inhibitors (ICIs). However, the frequent dysregulation of the CDK4/6-RB pathway and the inherently complex TIME in digestive cancers often precipitate primary and acquired drug resistance, which restricts their clinical efficacy. Consequently, elucidating the mechanisms that modulate drug sensitivity and developing biomarker-driven combination regimens have become critical research priorities. This review systematically summarizes the mechanisms governing the sensitivity of digestive tract tumors to CDK4/6 inhibitors. From the perspective of laboratory medicine, we further emphasize the importance of biomarker detection, therapeutic target assessment, and precision molecular subtyping in identifying patients most likely to benefit from CDK4/6 inhibitor-based therapies. In addition, we discuss the role of these agents in remodeling the TIME, evaluate current combination strategies aimed at overcoming resistance, and highlight future directions for advancing this rapidly evolving field.

## 1 Introduction

Dysregulation of the cell cycle is one of the most prominent biological hallmarks of malignant tumors (1). Ordered cell cycle progression depends on functional complexes formed by cyclins and cyclin dependent kinases, among which the Cyclin D1-CDK4/6-RB axis is the central pathway regulating the G1 to S phase transition and a key molecular foundation for sustained proliferation in many cancers (2). Under normal physiological conditions, Cyclin D1 binds to CDK4/6 to form an active complex, which in turn phosphorylates the RB. This phosphorylation disrupts the interaction between RB and E2F transcription factors, allowing E2F to activate the transcription of genes required for DNA replication and cell cycle progression, thereby driving entry into S phase (3). During tumorigenesis, this pathway is frequently maintained in a hyperactive state because of *CCND1* overexpression, aberrant CDK4/6 activation, or loss of RB function, ultimately leading to cell cycle dysregulation and uncontrolled tumor cell proliferation (3). On the basis of this mechanism, CDK4/6 inhibitors were developed to selectively block CDK4/6 activity, inhibit RB phosphorylation, and induce G1 arrest, thereby suppressing tumor cell proliferation and opening a new avenue for targeted cancer therapy (3–6).

Since their introduction into clinical practice, CDK4/6 inhibitors have been most extensively studied in breast cancer, where the supporting clinical evidence is also the strongest (7). In particular, CDK4/6 inhibitors have become an important component of systemic therapy for hormone receptor positive, human epidermal growth factor receptor 2 negative (HR+/HER2-) breast cancer (7). The currently approved agents, palbociclib, ribociclib, and abemaciclib, have all been incorporated into treatment strategies for HR+/HER2- breast cancer. Clinical studies have shown that, compared with endocrine therapy alone, the addition of CDK4/6 inhibitors significantly prolongs progression free survival and overall survival in patients with advanced breast cancer (8–10). As the field has evolved, combination approaches involving these agents have also shown promise in HER2 positive breast cancer and triple negative breast cancer. Experience in breast cancer has made it clear that the clinical value of CDK4/6 inhibitors extends beyond simple cell cycle blockade. Their importance also lies in their ability to be integrated with other therapeutic modalities and to contribute to patient selection, resistance monitoring, and treatment optimization. For these reasons, breast cancer has become the most mature solid tumor model for precision therapy based on CDK4/6 inhibition.

**Figure 1:**
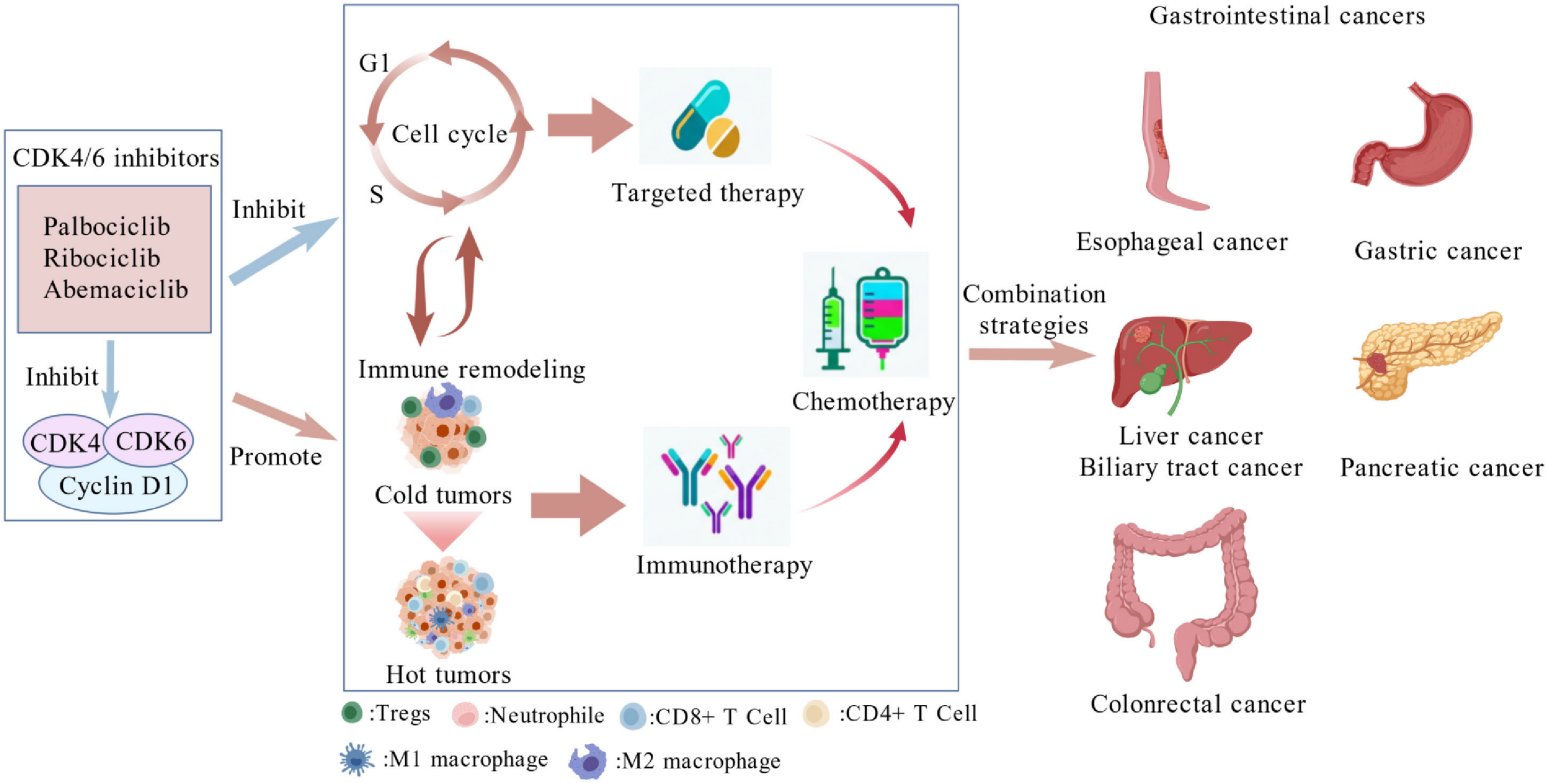
Mechanism and clinical applications of CDK4/6 inhibitors in gastrointestinal cancers. CDK4/6 inhibitors directly inhibit tumor cell proliferation. Meanwhile, by converting “cold tumors” into “hot tumors”, they reverse the immunosuppressive tumor microenvironment and synergize with targeted therapy, immunotherapy, and chemotherapy to improve therapeutic outcomes in various gastrointestinal cancers.

In contrast to the substantial benefit observed in breast cancer, the development of CDK4/6 inhibitors in gastrointestinal (GI) cancers has been relatively slow. GI cancers mainly include gastric cancer, colorectal cancer, esophageal cancer, pancreatic cancer, liver cancer, and biliary tract cancers (11). These tumors are characterized by high incidence and mortality, complex biology, and marked clinical heterogeneity (12, 13). Although aberrant activation of the Cyclin D1-CDK4/6-RB axis has also been observed in multiple GI cancers, providing a theoretical rationale for the use of CDK4/6 inhibitors, available evidence indicates that GI cancers differ substantially across primary sites, histologic subtypes, and molecular subgroups (12). In some tumors, proliferation is not primarily dependent on the Cyclin D1-CDK4/6-RB axis, but is also driven by alternative cell cycle regulatory networks such as the Cyclin E CDK2 pathway, which may attenuate the activity of CDK4/6 inhibitors (14). At the same time, both primary and acquired resistance to CDK4/6 inhibitors are prominent in GI cancers. Tumor cells can reactivate proliferative signaling through multiple bypass pathways, thereby restoring growth advantages and overcoming the cell cycle arrest induced by CDK4/6 inhibition. The tumor microenvironment in GI cancers further complicates treatment. A complex extracellular matrix and infiltration by immunosuppressive cell populations not only affect drug exposure, but also weaken antitumor immune responses (12). Lessons from breast cancer suggest that durable and stable clinical benefit from CDK4/6 inhibitors usually depends on rational combination strategies. To enhance drug sensitivity and delay the development of resistance, several combination approaches have been established around CDK4/6 inhibitors, including combinations with endocrine therapy to cooperatively suppress hormone receptor signaling, combinations with HER2 inhibitors, PI3K/AKT/mTOR inhibitors, and PARP inhibitors to block key bypass pathways and DNA damage repair mechanisms, and combinations with immune checkpoint inhibitors to enhance antitumor immune activity (5). A growing body of evidence also indicates that, beyond inducing cell cycle arrest, CDK4/6 inhibitors can increase tumor antigen presentation, modulate interferon related signaling, and remodel the tumor immune microenvironment, thereby improving tumor sensitivity to immunotherapy (15) (**Figure 1**).

Against this background, this review summarizes the current status and major challenges of CDK4/6 inhibitors in GI cancers, with a particular focus on resistance related pathways and remodeling of the tumor immune microenvironment. We also discuss, from the perspective of laboratory medicine, the potential value of biomarker testing, target assessment, and precision molecular subtyping in optimizing patient selection and supporting individualized treatment strategies, with the aim of providing a theoretical basis and clinical reference for the refinement of precision therapy in GI cancers.

## 2 Molecular determinants of CDK4/6 inhibitors sensitivity

### 2.1 The CDK4/6-RB pathway

In the search for targeted therapies for GI cancers, the cell cycle regulatory pathway centered on Cyclin D1-CDK4/6-RB-E2F has become a major focus. The precise operation of this pathway is essential for cells to transition from the G1 to the S phase and complete proliferation. Consequently, abnormalities in its components and functional status are implicated in the development and progression of nearly all GI cancers (16). During tumor development, the loss of RB protein function is a core event that drives cell cycle dysregulation and promotes malignant proliferation (17). This functional inactivation primarily occurs through hyperphosphorylation or ubiquitin-mediated degradation. Alternatively, genetic mutations can completely prevent the RB protein from being produced or functioning properly (18). RB dysregulation is particularly prominent in pancreatic and colorectal cancers, which are characterized by high malignancy and poor prognoses. Both cancers typically harbor activating mutations in the *KRAS* oncogene. This cooperation between tumor-initiating mutations and the loss of tumor suppressors further drives tumor proliferation and invasion (19, 20).

The deletion or mutation of endogenous CDK inhibitors is a major factor driving RB dysfunction, particularly involving the INK family (such as the p16 protein) and the CIP/KIP family (such as the p21 and p27 proteins) (21). In the majority of GI cancers, the *CDKN2A* gene, which encodes the INK family CDK4/6 inhibitor p16, frequently exhibits deletions or mutations (22–26). This genetic alteration prevents the normal expression of this natural, highly specific CDK4/6 inhibitors and abolishes its control over CDK4/6 activity. Consequently, the Cyclin D1-CDK4/6 complex remains persistently activated, driving the hyperphosphorylation of the RB protein and the subsequent loss of its tumor-suppressive function. This mechanistic shift renders GI cancers cells highly dependent on CDK4/6 for cell cycle progression, paradoxically enhancing their sensitivity to CDK4/6 inhibitors (27). This highlights the therapeutic potential of CDK4/6 inhibitors in these GI cancers. However, unlike p16, the CIP/KIP family proteins p21 and p27 exhibit dual roles: they act as CDK inhibitors but can also induce resistance to CDK4/6 inhibitors and promote tumor progression (28, 29). The transition of p27 from a traditional cell cycle repressor to a critical mediator of CDK4/6 inhibitors resistance fundamentally depends on the phosphorylation of its tyrosine 88 (Y88) residue (30). In *KRAS*-mutant colorectal cancer, Src family kinases are constitutively activated, which in turn catalyzes the phosphorylation of p27 at Y88. The binding mode of phosphorylated p27 (pY-p27) to the Cyclin D1-CDK4 complex undergoes a fundamental shift. Instead of inhibiting the complex, pY-p27 acts as an allosteric activator. The binding of pY-p27 induces a conformational rearrangement in the kinase domain of CDK4. This structural shift prevents inhibitors like palbociclib from binding effectively, thereby conferring drug resistance. In contrast to the phosphorylation-driven effects of p27, p21 induces cell proliferation primarily through its subcellular localization. In contexts with intact p53 function, DNA damage-induced upregulation of p21 effectively inhibits CDK2 activity, reduces RB phosphorylation levels, and blocks the E2F-mediated S-phase transcriptional program. Under these conditions, p21 functions as a “protective factor” for the RB pathway, maintaining the stable G1/S arrest induced by CDK4/6 inhibitors. In esophageal cancer, however, the abnormal cytoplasmic accumulation of p21 alters its functional properties. Cytoplasmic p21 no longer functions primarily as a CDK inhibitor. Instead, it binds to and stabilizes the Cyclin D1-CDK4 complex, promoting its assembly and structural integrity while simultaneously exerting anti-apoptotic effects by repressing apoptosis-related molecules (31). The regulation of CDK4/6 inhibitors sensitivity through the ubiquitin-mediated degradation of the RB protein has also been reported in GI cancers. For instance, Yu et al. (32) discovered that in colorectal cancer, FERM domain-containing protein 8 (FRMD8) is a scaffold-like protein implicated in protein–protein interactions and intracellular signaling regulation, and it exhibits high-affinity binding to the RB protein. By competitively blocking the binding of the E3 ligase MDM2 to RB, FRMD8 shields the protein from ubiquitination signals, drastically prolonging the half-life of the RB protein. Additionally, FRMD8 binds to CDK7, preventing it from activating CDK4 via phosphorylation at the threonine 172 residue. When colorectal cancer cells lack FRMD8, the unprotected RB protein is rapidly degraded by MDM2. Concurrently, CDK4 enters a hyperphosphorylated, highly active state, directly driving malignant transformation at the G1/S transition. Consequently, the presence of FRMD8 significantly enhances the therapeutic sensitivity of colorectal cancer cells to palbociclib.

Alterations in RB status are considered the core factor determining the efficacy of CDK4/6 inhibitors. However, abnormalities within the CDK4/6-RB pathway are not limited to RB itself. In GI cancers, this pathway frequently exhibits coordinated dysregulation across multiple nodes. Beyond the loss, inactivation, or persistent hyperphosphorylation of the RB protein, alterations in upstream cyclins, the activation of bypass pathways, and mutations in key tumor suppressor genes also profoundly influence the regulation of the G1/S transition and drug response.

*CCND1* is one of the most common driver alterations in GI cancers (33). Persistently elevated levels of Cyclin D1 induce a dependency on CDK4/6 activity in tumor cells. This dependency provides a strong theoretical rationale for CDK4/6 inhibitors therapy, yet it simultaneously increases the likelihood of compensatory bypass activation. The high frequency of *CCND1* amplification in GI cancers (34–36) leads to the constitutive activation of the Cyclin D1-CDK4 complex. For instance, Fang et al. (37) discovered that the expression of the E3 ubiquitin ligase MG53 is significantly downregulated in over 80% of tumor tissues from patients with colorectal and gastric cancers. Mechanistically, MG53 directly binds to the cyclin box domain of Cyclin D1 and catalyzes its K48-linked polyubiquitination, thereby promoting the proteasomal degradation of both Cyclin D1 and Cyclin D3. The knockout of the MG53 gene results in the massive accumulation of Cyclin D1 protein and subsequently accelerates tumor growth. Similarly, Zhao et al. (38) investigated FBXO4 in esophageal cancer, identifying it as the critical substrate recognition subunit of the SCF ubiquitin ligase complex that targets Cyclin D1. Their experiments demonstrated that FBXO4-deficient tumors exhibit concurrent Cyclin D1 overexpression, which paradoxically enhances the sensitivity of esophageal cancer to palbociclib. Beyond the Cyclin D1 pathway, RB can also be directly hyperphosphorylated by the Cyclin E-CDK2 complex to promote entry into the S phase. Crucially, the activity of this complex is independent of CDK4/6. Consequently, when Cyclin E is amplified or overexpressed, CDK2 can redundantly phosphorylate RB and bypass the G1 arrest, even if CDK4/6 is effectively suppressed by inhibitors (39). The abnormal upregulation of Cyclin E in gastric cancer has been identified as a primary driver of resistance to palbociclib (40).

In addition to these bypass mechanisms, the high-frequency mutation of the tumor suppressor gene *TP53* serves as a critical contributor to CDK4/6 inhibitors resistance. In hepatocellular carcinoma (HCC), the frequent occurrence of the *TP53* R249S mutation (41) establishes an aberrant, CDK4-dependent proliferation loop. This specific mutation not only abolishes the cell cycle inhibitory function of p53 but also reinforces proliferative signaling pathways by stabilizing CDK4. Studies indicate that CDK4 can phosphorylate the p53-R249S mutant, which subsequently promotes its binding to c-Myc. This interaction enhances the stability of Myc, ultimately driving the expression of cell cycle-related genes (42). Concurrently, in pancreatic cancer, functional missense mutations of *TP53*, specifically the p53-R273H/C mutants, have been shown to increase cell survival and confer profound resistance to palbociclib (43). In addition to canonical cell-cycle components, dynein light chain LC8-type 1 (DYNLL1) and interleukin enhancer-binding factor 2 (ILF2) may enhance CDK4 expression through post-transcriptional regulation, thereby increasing CDK4 dependency. By contrast, protein phosphatase 5 (PP5) may affect palbociclib sensitivity through modulation of AMPK activity, particularly in HCC (44, 45).

From a laboratory medicine perspective, biomarkers related to the CDK4/6–RB axis should be assessed within an integrated testing framework rather than interpreted on the basis of a single assay. Immunohistochemistry (IHC) remains the most practical first-line approach for evaluating protein-level alterations in formalin-fixed, paraffin-embedded (FFPE) tumor specimens, particularly for RB, p16, p21, p27, Cyclin D1, and p53. RB IHC is evaluated based on nuclear staining in tumor cells. Stromal cells, lymphocytes, endothelial cells, or adjacent non-neoplastic epithelial cells should serve as internal positive controls. RB expression is commonly assessed using the H-score system: H-score = 1 × percentage of weak staining + 2 × percentage of moderate staining + 3 × percentage of strong staining, with a total score ranging from 0 to 300 (46). Complete loss of nuclear RB staining in tumor cells, with preserved internal control staining, indicates RB protein loss. However, The reliability of IHC interpretation depends on multiple pre-analytical and analytical variables, including fixation quality, tissue processing, antibody clone and specificity, staining platform, scoring criteria, and intra-tumoral heterogeneity (47). For gene-level evaluation, fluorescence *in situ* hybridization (FISH) can provide single-cell resolution for copy-number alterations, such as *RB1* deletion, *CDKN2A* loss, and *CCND1* amplification. Targeted next-generation sequencing (NGS), in contrast, offers broader molecular profiling by detecting point mutations, small insertions and deletions, copy-number alterations, and coexisting pathway aberrations within the same assay. Nevertheless, NGS-based copy-number interpretation may be affected by tumor purity, sequencing depth, panel design, and bioinformatic algorithms (48). Therefore, discrepant or borderline findings should be interpreted in the context of tumor cellularity, histologic review. Liquid biopsy, particularly circulating tumor DNA (ctDNA) sequencing, provides a minimally invasive strategy for monitoring clonal evolution, acquired resistance, and emergent pathway alterations in advanced, metastatic, or unresectable disease. Its major advantage lies in dynamic longitudinal assessment, especially when repeat tissue biopsy is not feasible. Nevertheless, ctDNA results should not be regarded as a complete substitute for tissue-based testing. Assay sensitivity is strongly affected by tumor burden, anatomic disease distribution, ctDNA shedding rate, disease stage, sample input, and the limit of detection of the platform. False-negative results may occur, particularly in low-shedding tumors or when copy-number alterations are the primary alteration of interest. Accordingly, a non-informative ctDNA result should be interpreted with caution and, whenever possible, followed by tissue-based testing or orthogonal confirmation (49).

Although these biomarkers have clear biological relevance, their clinical implementation remains constrained by analytical and interpretative limitations. A key challenge is that molecular alteration does not always translate into functional pathway dependence. For example, RB protein loss may suggest resistance to CDK4/6 inhibition, but therapeutic response can still be modified by concurrent *TP53* mutation, PI3K/AKT/mTOR activation, MAPK signaling, or other bypass mechanisms. Similarly, *CCND1* overexpression or *CDKN2A* alteration does not necessarily indicate true CDK4/6 dependency. This explains why many biomarkers that perform well in preclinical models lose predictive power in heterogeneous clinical populations. p16 promoter methylation represents a typical example of this translational gap. Although p16 methylation is biologically linked to *CDKN2A* silencing and has been proposed as a promising biomarker, it has not become a robust clinical selection tool in gastrointestinal cancers. In addition, p16 methylation is not entirely tumor-specific and may be detected in premalignant or inflammatory gastrointestinal lesions, which reduces its specificity for clinical decision-making (50). Laboratory evaluation of CDK4/6–RB axis biomarkers should emphasize analytical validity, reproducibility, biological interpretability, and clinical utility. Future biomarker strategies should move from single-marker detection toward integrated multi-marker models combining IHC, FISH, NGS, methylation-specific assays, and ctDNA-based longitudinal monitoring. More importantly, these laboratory markers need to be validated in prospective biomarker-enriched clinical trials before they can be reliably used for patient selection, resistance monitoring, or treatment adjustment in GI cancers.

### 2.2 Synergistic signaling pathways

**Figure 2:**
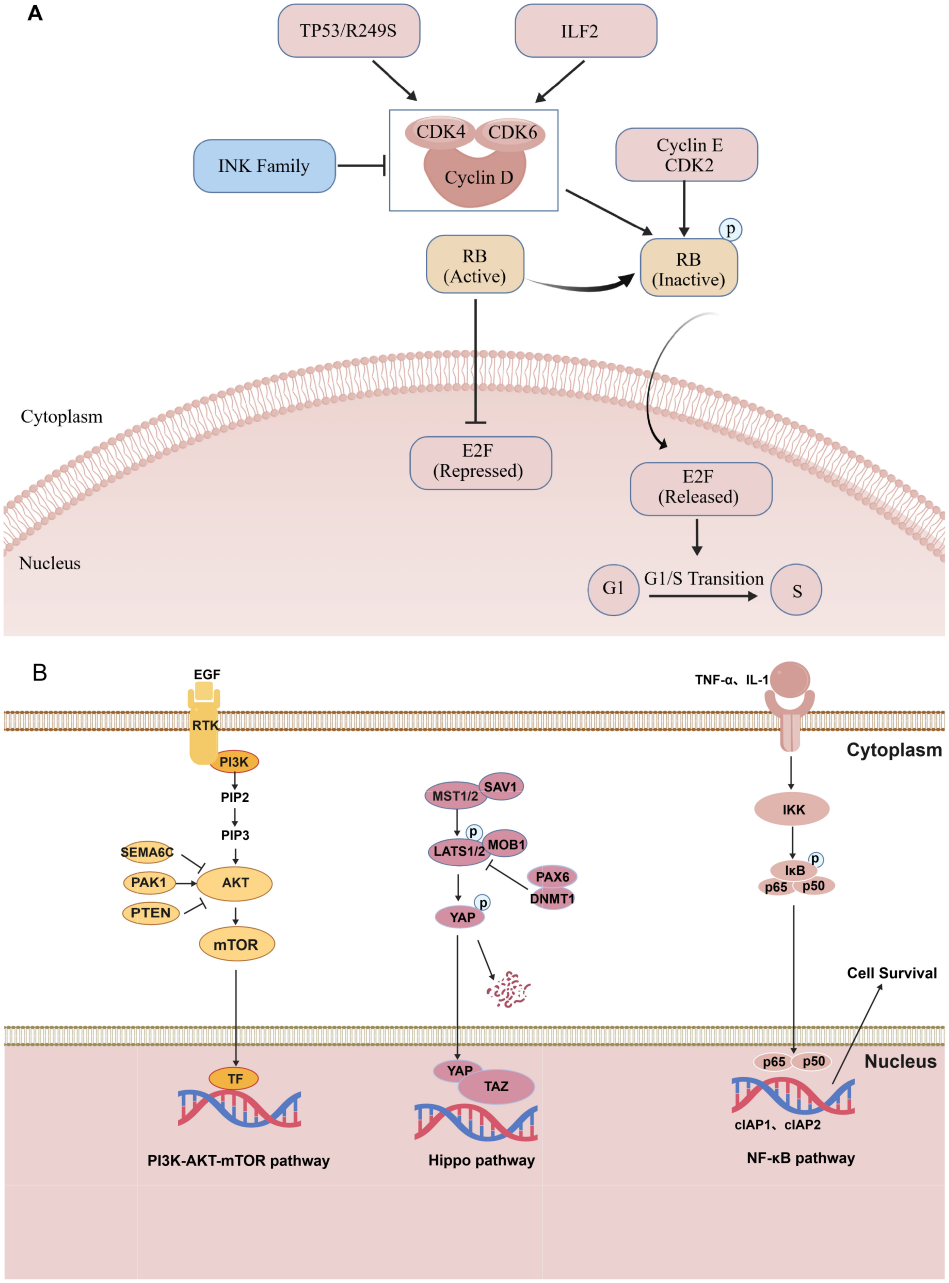
Core cell cycle pathway and synergistic signaling pathways. **(A)**: Schematic of the canonical CDK4/6-RB-E2F pathway regulating the G1/S transition. **(B)**: Overview of representative synergistic signaling pathways, including PI3K-AKT-mTOR, Hippo-YAP, and NF-κB pathways.

The core mechanism of action for CDK4/6 inhibitors relies on blocking the phosphorylation of the RB protein, thereby inducing cell cycle arrest in the G1 phase. While the integrity of the RB pathway is crucial for the clinical application of CDK4/6 inhibitors in solid tumors, the efficacy of monotherapy remains limited even in tumors with intact RB expression. This limitation arises because, beyond the canonical RB signaling axis, GI cancers harbor complex, synergistic signaling networks capable of bypassing or counteracting the cell cycle arrest induced by CDK4/6 blockade (11). In GI cancers, alongside cell cycle dysregulation, multiple synergistic signaling pathways exhibit high mutation frequencies or aberrant expression across various tumor types. For instance, *KRAS* mutations are present in 40%~50% of colorectal cancers (51) and 70%~90% of pancreatic cancers (52), leading to the loss of intrinsic GTPase activity. Furthermore, alterations in the PI3K/AKT pathway, including *PIK3CA* mutations, PTEN loss, and AKT hyperactivation, occur frequently in various GI cancers, robustly promoting tumor cell proliferation and survival (53) (**Figure 2**).

#### 2.2.1 PI3K/AKT/mTOR signaling pathway

Investigations into how mutations within the PI3K/AKT/mTOR signaling pathway alter sensitivity to CDK4/6 inhibitors primarily focus on the consequences of pathway hyperactivation. For instance, Hung et al. (54) investigated pancreatic cancer, where the expression of semaphorin 6C (SEMA6C) is frequently downregulated via the targeting of microRNA miR-124-3p. Their research demonstrates that SEMA6C deficiency abolishes the intrinsic suppression of the AKT pathway, leading to elevated AKT phosphorylation. Activated AKT subsequently phosphorylates and inhibits GSK3, thereby preventing the degradation of β-catenin. The nuclear accumulation of β-catenin acts as a transcription factor to drastically upregulate both the mRNA and protein levels of Cyclin D1. Driven by this profound dependency on *CCND1* overexpression, treatment with the CDK4/6 inhibitor palbociclib significantly suppresses the proliferation of SEMA6C-low pancreatic cancer cells in a dose-dependent manner. In the context of gastric cancer, another major GI cancer, Qian et al. (55) revealed the regulatory role of p21-activated kinase 1 (PAK1) in dictating CDK4/6 inhibitors sensitivity. As an effector molecule of the Rho family GTPases Cdc42 and Rac1, PAK1 actively participates in tumor progression by modulating cytoskeletal reorganization, cell motility, and survival signaling. Treatment with palbociclib reduces the viability of gastric cancer cells (SGC-7901 and MKN-45) while concurrently downregulating PAK1 expression. The silencing of PAK1 significantly enhances the sensitivity of these GI tumor cells to CDK4/6 inhibitors and induces substantial DNA damage, suggesting that PAK1 may serve as a novel therapeutic target to overcome CDK4/6 inhibitors resistance. The loss of function of *PTEN*, a critical negative regulator of the PI3K/AKT pathway, serves as another major driver of constitutive AKT activation (56). Lim et al. (57) explored resistance mechanisms and potential overcoming strategies by establishing patient-derived cell (PDC) models from metastatic colorectal cancer harboring *BRAF* and *PTEN* co-mutations. For *BRAF* V600E-mutant colorectal cancer, the combination of encorafenib and cetuximab represents a standard second-line therapy. However, PDC models carrying both the *BRAF* V600E mutation and the *PTEN* R173C loss-of-function mutation exhibit profound resistance to this regimen. Notably, the addition of the CDK4/6 inhibitor ribociclib to form a triplet therapy with encorafenib and cetuximab significantly inhibits cell proliferation, demonstrating a robust synergistic effect. To counteract the hyperactivation of AKT signaling, specific non-coding RNAs exert potent suppressive effects. Callegari et al. (58) discovered that microRNA-199a-3p is significantly downregulated in HCC. Restoring its expression effectively suppresses the PI3K/AKT pathway by directly targeting mTOR and PAK4. Although palbociclib monotherapy successfully inhibits RB phosphorylation, it concurrently induces compensatory AKT activation, a response that may severely restrict its overall therapeutic efficacy. Experimental treatment with miR-199a-3p mimics completely abrogates this aberrant AKT activation and significantly enhances tumor cell apoptosis, proving highly effective even in sorafenib-resistant cells. In murine models of sorafenib-resistant liver cancer, the overexpression of miR-199a-3p remarkably amplifies the efficacy of palbociclib, providing a novel paradigm to overcome drug resistance. Similarly, Ronny et al. (59) investigated the non-coding RNA nc886 in esophageal squamous cell carcinoma, a highly aggressive GI cancer. This RNA is frequently silenced through promoter CpG methylation. The silencing of nc886 triggers the aberrant activation of the AKT pathway, leading to the downregulation of the CDK inhibitors *CDKN2A* and *CDKN2C*, alongside the direct upregulation of CDK4. Esophageal cancer cells lacking nc886 exhibit exceptionally active G1~S phase transitions. Because these cells become highly dependent on overexpressed CDK4 to drive the cell cycle, they demonstrate remarkable sensitivity to palbociclib.

#### 2.2.2 Hippo signaling pathway

Beyond the PI3K/AKT/mTOR pathway, other signaling pathways have also been reported to modulate the sensitivity of GI cancers to CDK4/6 inhibitors. Among them, the Hippo pathway plays a key tumor-suppressive role. Its core components include the MST1/2 kinases, the LATS1/2 kinases, and the downstream effectors YAP and TAZ. When the Hippo pathway is activated, MST1/2 phosphorylates and activates LATS1/2, which subsequently phosphorylate YAP/TAZ (60). In contrast, when the Hippo pathway is inactivated, unphosphorylated YAP/TAZ translocate into the nucleus and bind to TEAD family transcription factors, thereby activating target gene transcription and promoting cell proliferation and invasion. The Hippo pathway is closely linked to cell cycle control. Nuclear YAP/TAZ can directly upregulate *CCND1*, *CCNE1*, and *CDK6*, thereby enhancing CDK4/6 complex activity and accelerating the G1/S transition (61). Through the regulation of Cyclin D1 and CDK6, Hippo-YAP signaling influences the sensitivity of GI cancers to CDK4/6 inhibitors. Dhir et al. (62) reported that YAP1 is highly active in pancreatic cancer cells, accompanied by elevated expression of its target genes *CDK6* and *CCND1*. *YAP1* knockdown increased the sensitivity of pancreatic cancer cells to abemaciclib. Zhang et al. (63) found that the transcription factor PAX6 is highly expressed in gastric cancer. PAX6 interacts with DNA methyltransferase 1 (DNMT1), stabilizing the DNMT1 protein and increasing its enzymatic activity. Enhanced DNMT1 activity leads to hypermethylation and transcriptional silencing of the *LATS2* promoter, and the resulting reduction in *LATS2* expression causes Hippo pathway inactivation. In colorectal cancer, Wen et al. (64) further showed that abemaciclib can also block the nuclear translocation of YAP. Mechanistically, CDK4/6 phosphorylates deubiquitinating enzyme 3 (DUB3), and activated DUB3 removes the ubiquitin mark from YAP1, thereby preventing its proteasomal degradation.

#### 2.2.3 NF-κB signaling pathway

The NF-κB signaling pathway is a central regulator of cell survival, inflammation, and immune responses (65). Under resting conditions, NF-κB dimers are retained in the cytoplasm through binding to the inhibitory protein IκB. Upon stimulation by factors such as TNF-α and IL-1, the IκB kinase complex becomes activated and phosphorylates IκB, leading to its ubiquitin-mediated degradation. This process releases NF-κB, allowing its nuclear translocation and subsequent activation of target gene transcription. NF-κB target genes include a wide range of anti-apoptotic proteins, pro-inflammatory cytokines, chemokines, and immunoregulatory molecules (66). Among them, cIAP1 and cIAP2 are members of the inhibitor of apoptosis protein family and serve important regulatory roles in signaling pathways related to cell death and survival by suppressing apoptotic signaling (67). Menapree et al. (68) revealed that the protein levels of NF-κB downstream effectors cIAP1/2 are significantly elevated in cholangiocarcinoma tissues compared with paired adjacent normal tissues. The therapeutic efficacy of palbociclib monotherapy is limited in cholangiocarcinoma cells, and one important reason is the activation of the NF-κB pathway, which leads to the upregulation of cIAP1/2. Palbociclib activates NF-κB signaling by inducing p65 phosphorylation and nuclear translocation. This effect may be related to the reduced inhibitory effect of RB on p65 following palbociclib-mediated suppression of RB phosphorylation.

#### 2.2.4 Notch signaling pathway

The Notch signaling pathway plays a critical role in cell proliferation and apoptosis. Upon binding of NOTCH receptors (NOTCH1–4) to their ligands (JAGGED1/2 and DLL1/3/4), the Notch intracellular domain (ICD) is released and translocates to the nucleus, where it forms a transcriptional activation complex with RBP-J/CSL and MAML1 to activate downstream target genes (69). In addition to canonical components of the Notch pathways, A recent study has identified Proline-rich homeodomain protein/Hematopoietically expressed homeobox (PRH/HHEX) as an important upstream transcriptional regulator involved in cholangiocarcinoma progression. PRH/HHEX is a homeobox transcription factor that can regulate target gene transcription through DNA binding via its homeodomain and can also modulate the activity of other transcriptional regulators through protein-protein interactions (70). Kitchen et al. (71) found that *HHEX* mRNA expression was significantly higher in cholangiocarcinoma tissues than in matched adjacent bile duct tissues. PRH upregulates Cyclin D2 through NOTCH3 and directly activates Wnt pathway genes such as *WNT11* and *WNT16*, thereby enhancing Wnt signaling and increasing *CCND1* expression. At the same time, PRH directly suppresses the expression of p15 (*CDKN2B*) and p27 (*CDKN1B*). As a result, cholangiocarcinoma cells with high PRH expression become highly dependent on CDK4/6 activity, which markedly increases their sensitivity to palbociclib.

These signaling pathways are usually evaluated by combining protein-level and gene-level. For the PI3K/AKT/mTOR pathway, IHC can be used to assess pathway activation markers such as p-AKT, p-mTOR, p-S6 and p-4EBP1 (72). However, phosphorylated proteins are sensitive to tissue fixation and pre-analytical handling, so IHC results should be interpreted cautiously and correlated with genomic findings. Targeted NGS may further identify sequence variants or copy-number alterations involving *PIK3CA*, *PTEN*, *AKT1*, *MTOR* and related genes; Hippo pathway activity is commonly inferred from the expression and subcellular localization of its key transcriptional coactivators, YAP and TAZ. Cytoplasmic retention of YAP/TAZ, often associated with canonical Hippo pathway activation, suggests suppression of YAP/TAZ-mediated transcriptional activity, whereas nuclear accumulation indicates active YAP/TAZ-dependent transcription of downstream target genes. Because YAP/TAZ localization may also be regulated by non-canonical, mechanical, metabolic and tumor microenvironmental signals, nuclear YAP/TAZ staining should be interpreted as evidence of YAP/TAZ activation rather than as a stand-alone marker of Hippo pathway gene inactivation (73); NF-κB pathway activity is commonly inferred from p65/RelA nuclear localization or phosphorylated p65 expression by IHC. Loss of IκBα or increased inflammatory mediators, such as IL-6 and IL-8, may provide additional supporting evidence. Nevertheless, NF-κB activation is not tumor-specific and may also reflect inflammation, infection, necrosis or treatment-related stress, which reduces its specificity as a clinical biomarker (74); Notch signaling can be evaluated by integrating protein-level, genomic and transcript-level assays. IHC may be used to assess NOTCH receptors, mainly NOTCH1–4, ligands such as JAGGED1 and DLL family members, and downstream effectors such as HES1 and HEY1. qPCR, RNA sequencing or mRNA *in situ* hybridization can provide additional evidence of pathway transcriptional activity (75).

Although the preceding sections focused on canonical resistance mechanisms, accumulating evidence indicates that CDK4/6 inhibition does not merely block proliferation but also actively reprograms the tumor immune microenvironment through the RB-E2F axis. Under CDK4/6 inhibition, hypophosphorylated RB binds and represses E2F transcription factors, thereby downregulating not only cell-cycle-related genes but also a set of immune-related E2F target genes, including chemokines and antigen presentation machinery components. Furthermore, sustained cell cycle arrest can trigger senescence, and senescent tumor cells acquire the senescence-associated secretory phenotype (SASP), which secretes a dynamic array of cytokines, growth factors, and proteases (16). The p53-p21-RB pathway similarly couples oncogenic stress and DNA damage responses to cell-cycle arrest while modulating cytokine output, immune surveillance, and PD-1/PD-L1-related signaling (76). Accordingly, RB, p53, and E2F should be viewed not only as cell-cycle regulators, but also as central mediators of tumor-immune crosstalk. This framework helps explain why CDK4/6 inhibitors exert immunomodulatory effects that extend beyond cytostatic arrest alone.

## 3 Effects of CDK4/6 inhibitors on tumor immune microenvironment remodeling

**Figure 3:**
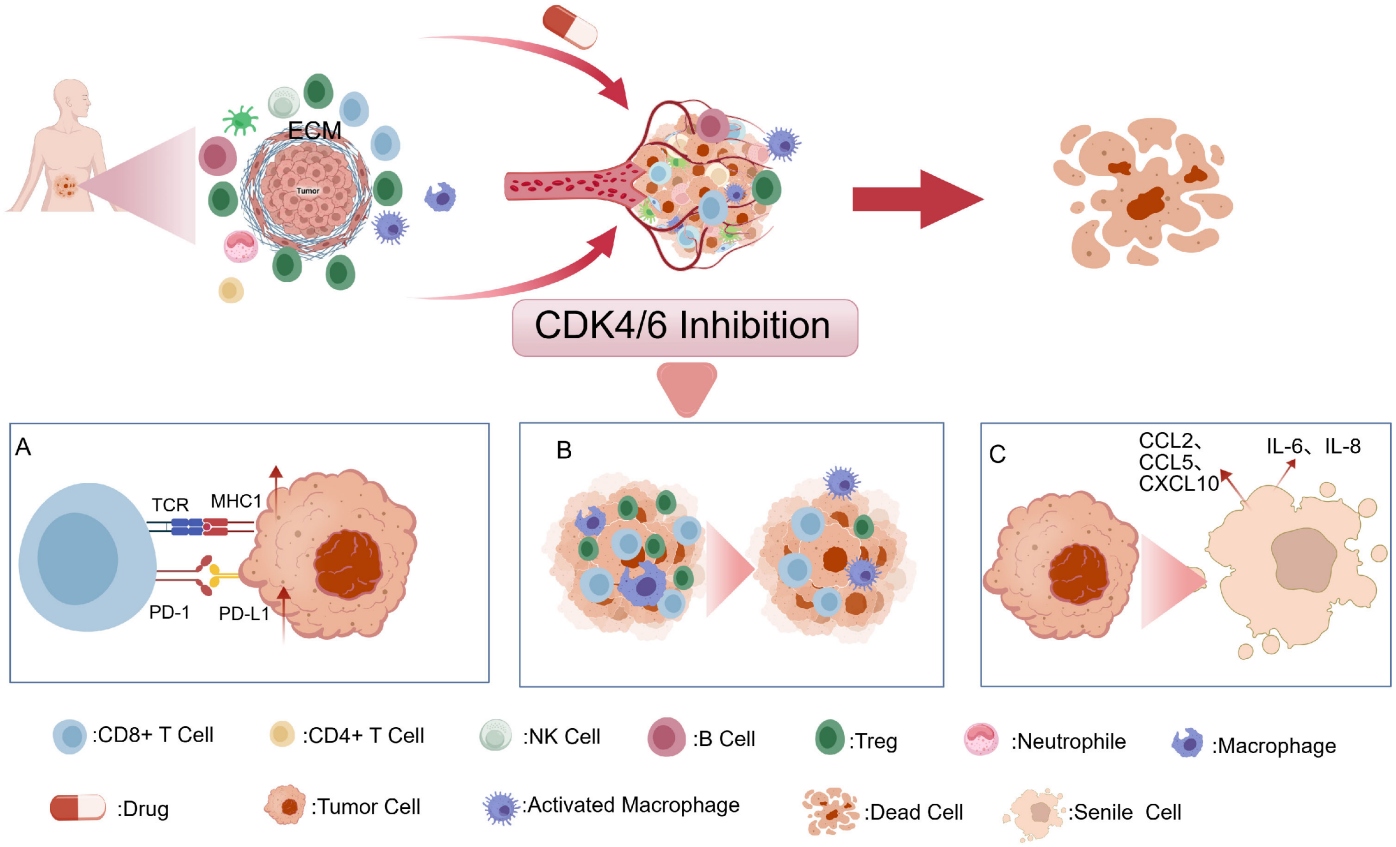
Immunomodulatory effects of CDK4/6 inhibition in the tumor microenvironment. Treatment with CDK4/6 inhibitors induces tumor cell death and promotes remodeling of the tumor microenvironment. **(A)**: Enhanced antigen presentation by tumor cells and improved T-cell recognition, accompanied by modulation of the PD-1/PD-L1 immune checkpoint axis. **(B)**: Altered composition of immune cell infiltration in tumor tissues, which boosts antitumor immunity. **(C)**: Induction of the senescence-associated secretory phenotype in tumor cells, thereby regulating the release of cytokines and chemokines.

Remodeling the tumor immune microenvironment is considered one of the key antitumor strategies. Critical determinants of the tumor immune microenvironment include immune cell infiltration, the secretion of cytokines and chemokines, and the expression of immune checkpoint molecules. In GI cancers, the immune microenvironment generally exhibits marked immunosuppressive features (77). Regulatory T cells (Tregs), which suppress antitumor immune responses, are significantly enriched in tumor tissues (78). Tumor-associated macrophages (TAMs) are abundant and are predominantly polarized toward the immunosuppressive M2 phenotype, while myeloid-derived suppressor cells (MDSCs) accumulate in large numbers and inhibit the function of effector T cells (77). In addition, cancer-associated fibroblasts (CAFs) and the extracellular matrix (ECM) form a dense stromal structure that not only secretes multiple immunoregulatory factors to sustain an immunosuppressive environment but also creates a physical barrier that limits immune cell infiltration into the tumor core (79) (**Figure 3**).

### 3.1 Enhancement of tumor antigen presentation and “viral mimicry”

With increasing investigation of CDK4/6 inhibitors, it has become clear that, beyond regulating the CDK4/6-RB-E2F signaling axis, these agents also exert beneficial effects on the tumor immune microenvironment. It has now been demonstrated that CDK4/6 inhibitors can enhance tumor antigen presentation. Goel et al. (80) reported that treatment of breast cancer cells with the abemaciclib activated the expression of endogenous retroviral elements (ERVs) and enhanced tumor antigen presentation, a phenomenon referred to as the “viral mimicry” effect. Mechanistically, this effect is mediated by downregulation of the DNMT1, which reduces methylation levels in the promoter regions of endogenous retroviral genes and thereby relieves their transcriptional repression. This process induces the production of type III interferon (IFN-λ), promotes tumor antigen presentation, and strengthens tumor-specific T cell responses. This mechanism is particularly relevant because reduced antigen presentation is a well-recognized mode of immune escape in cancer (81). By restoring expression of antigen-processing machinery, CDK4/6 inhibitors may counteract one of the central barriers limiting T-cell-mediated tumor clearance. More broadly, recent reviews have emphasized that the immunologic consequences of ERV reactivation are not limited to interferon induction; derepressed retroviral transcripts and their derived peptides may also contribute to enhanced tumor immunogenicity by broadening the repertoire of antigens available for immune recognition. These observations support the concept that “viral mimicry” is not merely a transcriptional phenomenon, but a functional bridge linking epigenetic reprogramming to improved antigen presentation and immune activation (82). It remains noteworthy that *DNMT1* overexpression is common in gastric cancer, pancreatic cancer, and hepatocellular carcinoma, and that *DNMT1* downregulation can increase E-cadherin expression and thereby suppress tumor cell migration and invasion (83–85). The induction of viral mimicry by CDK4/6 inhibitors has been linked to the downregulation of *DNMT1*. Notably, suppression of DNMT1 protein has been demonstrated to induce viral mimicry in colorectal cancer and subsequent immune activation in this tumor type, providing direct mechanistic support for this pathway (86, 87). Roulois et al. (87) demonstrated that low-dose the DNMT1 inhibitor 5-AZA-CdR targets colorectal cancer-initiating cells by inducing viral mimicry. Nevertheless, direct validations based on clinical colorectal cancer specimens are still lacking. Furthermore, DNA methyltransferase inhibitors targeting DNMT1 are currently under clinical evaluation in colorectal cancer (88). Schaer et al. (89) further investigated the immunomodulatory effects of abemaciclib in colorectal cancer models and found that CDK4/6 inhibition could also enhance tumor antigen presentation by increasing MHC-I and MHC-II expression on tumor cells and macrophages, as well as MHC-I expression on dendritic cells.

### 3.2 Modulation of immune cell infiltration

CDK4/6 inhibition can act on immune-infiltrating cells within the tumor microenvironment and enhance the infiltration of effector immune cells. CDK4/6 inhibitors suppress the proliferation of regulatory T cells (Tregs). In contrast, cytotoxic CD8+ T cells are less affected by CDK4/6 inhibition, thereby reducing the intratumoral Treg/CD8+ T-cell ratio (89). In addition, CDK4/6 inhibitors can enhance intratumoral CD8+ T-cell infiltration through functional reprogramming of tumor-associated macrophages (TAMs), thereby promoting indirect interactions between tumor cells and CD8+ T cells and strengthening the cytotoxic activity of CD8+ T cells against tumor cells (90). CDK4/6 inhibitors can also activate T cells through the NFAT signaling pathway. NFAT4, a member of the NFAT transcription factor family, is involved in T-cell activation and regulates the expression of multiple immune-related genes. NFAT4 can be phosphorylated by Cyclin D1-CDK6, whereas CDK4/6 inhibitors reduce NFAT phosphorylation and enhance its transcriptional activity. This leads to the upregulation of NFAT target genes, thereby promoting T-cell activation and strengthening antitumor immune responses. CDK4/6 inhibitors can also increase the infiltration of effector T cells into tumors, which may be related to the elevated levels of chemokines such as CXCL9 and CXCL10 following CDK4/6 inhibitors treatment (91).

### 3.3 Regulation of PD-L1 expression and immune checkpoint axis

CDK4/6 inhibitors also modulate the tumor immune microenvironment through regulation of PD-L1 expression and the broader immune checkpoint axis. This is particularly important because PD-L1 is a central mediator of tumor immune escape, and its expression is dynamically controlled by oncogenic signaling, inflammatory stimuli, and post-translational regulatory mechanisms. Accordingly, the CDK4/6-RB pathway has emerged as an important molecular interface linking cell-cycle control to immune checkpoint regulation (92).

Zhang et al. (93) reported that CDK4/6 inhibitors promote the ubiquitination and degradation of PD-L1 by reducing the phosphorylation level of SPOP. Jin et al. (94) further showed that the Cyclin D1-CDK4/6 complex promotes the binding of RB to the NF-κB p65 subunit through phosphorylation of RB at S249/T252, thereby suppressing PD-L1. CDK4/6 inhibitors disrupt this process and consequently upregulate PD-L1 expression. Together, these findings provide a theoretical basis for combining CDK4/6 inhibitors with PD-1/PD-L1 blockade. From a therapeutic perspective, these findings provide a strong mechanistic rationale for combining CDK4/6 inhibitors with PD-1/PD-L1 blockade. By increasing tumor immunogenicity and effector immune activity while simultaneously inducing adaptive checkpoint signaling, CDK4/6 inhibition may create a tumor state that is more dependent on PD-1/PD-L1-mediated immune suppression and therefore more vulnerable to checkpoint blockade. Preclinical studies have accordingly shown that CDK4/6 inhibitors can synergize with anti-PD-1 or anti-PD-L1 therapy to enhance antitumor efficacy. Although this strategy still requires further validation in GI cancers, the available data support PD-L1 regulation as an important mechanistic basis for such combination approaches.

### 3.4 Induction of senescence-associated secretory phenotype

CDK4/6 inhibitors can also remodel the tumor immune microenvironment through induction of cellular senescence and the subsequent development of SASP (95, 96). Senescent cells secrete pro-inflammatory factors such as IL-6 and IL-8, chemokines including CCL2 and CXCL10, and various growth factors (92). These factors can recruit and activate immune cells, thereby enhancing antitumor immune responses.

Evidence supporting this mechanism has been reported in gastrointestinal tumor models. In HCC, palbociclib induces senescence in hepatic tumor-initiating cells, and these senescent cells secrete SASP factors that recruit and activate immune populations, thereby enhancing immune-mediated tumor suppression and inhibiting hepatocarcinogenesis. Mechanistically, CDK4/6 inhibition promotes cytosolic DNA accumulation, which is sensed by DEAD-box helicase 41 (DDX41), a cytosolic DNA sensor induced under hypoxic conditions in the tumor microenvironment. Activation of the DDX41–STING axis subsequently amplifies inflammatory SASP production, indicating that CDK4/6 inhibitor-induced senescence is not merely a cell-autonomous growth-arrest program but also engages innate immune surveillance within the liver tumor microenvironment (97). This finding suggests that CDK4/6 inhibitors-induced senescence is not merely a cell-autonomous growth arrest program, but can also engage innate immune mechanisms that contribute to tumor suppression in the liver microenvironment. A related phenomenon has been observed in colorectal cancer models, in which CDK4/6 inhibition can trigger inflammatory signaling programs linked to the cGAS-STING pathway. Activation of cGAS-STING in colorectal cancer is known to induce type I interferons and inflammatory chemokines such as CCL5 and CXCL10, both of which promote antitumor immune activation and recruitment of cytotoxic lymphocytes (98). More broadly, recent work indicates that CDK4 or CDK6 blockade can generate endogenous DNA damage and activate STING-dependent type I interferon responses. In this setting, CDK4/6 inhibitors-driven senescence may contribute to increased infiltration of CD8+ T cells and NK cells while reducing immunosuppressive myeloid populations, thereby shifting the tumor microenvironment toward a more immune-reactive state.

## 4 Biomarker-guided combination strategies for CDK4/6 inhibitors

Long-term monotherapy with CDK4/6 inhibitors frequently leads to drug resistance through aberrant activation of compensatory oncogenic signaling pathways. Dysregulated pathways such as the Hippo-YAP/TAZ and PI3K/AKT/mTOR axes reinitiate cell cycle progression, sustain cancer cell stemness, and enable evasion of apoptosis, thereby blunting the CDK4/6 inhibition-induced blockade of the RB-E2F cascade. Such remodeling of resistance-associated signaling networks fundamentally alters tumor drug sensitivity and malignant phenotypes, which has reshaped clinical therapeutic strategies from single-agent suppression toward combinatorial multi-target intervention. Dual blockade of CDK4/6 signaling together with core resistance pathways effectively abrogates compensatory pathway activation and reverses drug resistance, providing a solid mechanistic rationale for the design of CDK4/6 inhibitor-based combination regimens.

Although CDK4/6 inhibitors have not yet been approved for GI cancers, growing evidence indicates that they can act synergistically with multiple classes of anticancer agents. Given the molecular heterogeneity and pathway alterations that characterize different GI cancers, biomarker-guided combination strategies may improve therapeutic efficacy by enabling coordinated pathway suppression, overcoming adaptive resistance, and remodeling the tumor immune microenvironment. Current combination approaches involve clinically used targeted agents, chemotherapeutic drugs, and immunotherapies, as well as emerging inhibitors that remain at the preclinical stage but have shown promise when combined with CDK4/6 inhibitors (**Table 1**).

**Table 1:** Biomarker-guided combination strategies of CDK4/6 inhibitors in gastrointestinal cancers.

Cancer	Drug category	Agents	Clinical stage	References
Esophageal cancer	PI3Kα inhibitor	CYH33, Alpelisib	Preclinical	(99)
	EGFR inhibitor	Erlotinib, Afatinib	Preclinical Clinical trial (II)	(100)
	Hippo/YAP1 inhibitor	Verteporfin, CA3	Preclinical	(101)
	Glutaminase inhibitor	CB-839	Preclinical	(102)
	Chemotherapy	Paclitaxel, Cisplatin	Preclinical	(35)
Gastric cancer	DNA methylation inhibitor	5-Aza	Preclinical	(63)
	eIF4E inhibitor	Ribavirin	Preclinical	(103)
	Tyrosine kinase inhibitors	Pyrotinib	Clinical trial (I)	(104)
	FAK inhibitor	FAK inhibitor	Preclinical	(105)
	BRD4 inhibitor	JQ1	Preclinical	(106)
Liver cancer	PI3K/AKT/mTOR inhibitor	Alpelisib, MK-2206, TAK228	Preclinical	(107)
	FGFR inhibitor	Infigratinib	Preclinical	(108)
	IκB kinase β inhibitor	Bay 11-7082	Preclinical	(109)
	Multikinase inhibitor	Sorafenib, Regorafenib	Preclinical	(110, 111)
	Mutant p53 reactivator	CP-31398	Preclinical	(42)
	Nuclear export inhibitor	KPT-330	Preclinical	(112)
Biliary tract cancer	pan-mTOR inhibitor	MLN0128	Preclinical	(113)
	FAK inhibitor	PND1186	Preclinical	(114)
	cIAP1/2 antagonist	LCL161	Preclinical	(68)
	Chemotherapy	Gemcitabine, Cisplatin	Preclinical	(115)
Pancreatic cancer	ERK inhibitor	Ulixertinib	Clinical trial (I)	(116)
	IGF1R/IR inhibitor	BMS-754807	Preclinical	(117)
	MEK inhibitor	Trametinib	Clinical trial (I)	(96, 118–120)
	mTOR inhibitor	Everolimus	Clinical trial (I)	(121)
	HCQ	HCQ	Retrospective analysis	(122)
	Hippo/YAP1 inhibitor	Verteporfin, CA3, ELAVL1	Preclinical	(62)
	TGF-β receptor inhibitor	SB-505124	Preclinical	(123)
	Multikinase inhibitor	Sorafenib	Preclinical	(124)
	Chemotherapy	Paclitaxel, Gemcitabine,	Clinical trial (Ib)/Preclinical	(125–127)
	Immunotherapy	Anti−PD−L1	Preclinical	(19)
Colorectal cancer	PI3K/AKT/mTOR inhibitor	Alpelisib, Gedatolisib	Preclinical	(128, 129)
	MEK inhibitor	PD0325901, Trametinib, Binimetinib	Preclinical Clinical trial (I) Clinical trial (II)	(119, 129–131)
	pan-RAF inhibitor	LY3009120	Preclinical	(132)
	BRAF/EGFR-targeted combination	Encorafenib, Cetuximab	Preclinical Clinical trial (II)	(57, 133)
	FRMD8-derived peptide	LKCHE motif	Preclinical	(32)
	HSP90 inhibitor	Ganetespib	Preclinical	(134)
	CDC37 inhibitor	DDO-6079	Preclinical	(135)
	Tankyrase inhibitor	MSC2504877	Preclinical	(136)
	Src inhibitor	Saracatinib	Preclinical	(30)
	PARP inhibitor	Talazoparib	Preclinical	(98)
	CD73 inhibitor	AB680	Preclinical	(137)
	Chemotherapy	Irinotecan	Preclinical	(138)
	Immunotherapy	P-PBA/PLL (IL-2), Anti−PD1	Preclinical	(139, 140)

### 4.1 Targeting PI3K/AKT/mTOR, MAPK pathway and upstream RTKs

The PI3K/AKT/mTOR pathway is one of the most frequently altered signaling nodes in GI cancers, driving *CCND1* expression and sustaining cell cycle progression even under CDK4/6 blockade. In esophageal cancer and colorectal cancer, selective PI3Kα inhibitors such as CYH33 and alpelisib have been integrated with palbociclib to robustly suppress survival signaling and sensitize tumors to cell cycle arrest (99, 128). A similar strategy has been validated in HCC, where combining palbociclib with PI3K/AKT/mTOR inhibitors (alpelisib, MK-2206, and TAK228) significantly reduces p-Rb levels and reinforces G1 phase transition delay (107). Beyond these, biliary tract cancer also benefits from such co-targeting: palbociclib synergizes with the pan-mTOR inhibitor MLN0128 (sapanisertib) by reciprocally enhancing mTOR blockade while suppressing compensatory *CCND1* upregulation (113).

Horizontal signaling through the MAPK pathway frequently compensates for CDK4/6 inhibition, leading to treatment failure. Evidence from pancreatic cancer demonstrates that the ERK inhibitor ulixertinib and the MEK inhibitor trametinib each exhibit potent synergy with palbociclib, an effect that is particularly pronounced in the adenosquamous subtype (118). This cooperative interaction extends to colorectal cancer, where trametinib, PD0325901 or the pan-RAF inhibitor LY3009120 prevents the emergence of resistant clones driven by compensatory MAPK reactivation (129, 130, 132). Beyond the downstream signaling cascades, upstream receptor tyrosine kinases act as key regulators that activate both the PI3K/AKT/mTOR and MAPK pathways, representing an additional rationale for combining CDK4/6 inhibitors with RTK-targeted therapies. For esophageal squamous cell carcinoma, dual blockade of EGFR (using erlotinib or afatinib) and CDK4/6 has been proposed to address the frequent coexistence of cell cycle dysregulation and receptor tyrosine kinase dependence (100). In pancreatic cancer, the IGF1R/IR inhibitor BMS-754807 cooperates with palbociclib to promote apoptosis (116, 117). Notably, the triplet regimen of ribociclib, the BRAF inhibitor encorafenib, and the EGFR inhibitor cetuximab is essential for treating *BRAF*-mutant colorectal cancer with *PTEN* loss, as it effectively blocks multiple escape routes to achieve responses (57).

### 4.2 Modulation of the Hippo and NF-κB pathway

Aberrant YAP1 activation can bypass CDK4/6 dependence by driving the transcription of various cyclins. Targeting this axis with verteporfin or CA3 enhances the efficacy of abemaciclib in esophageal cancer and pancreatic cancer (62, 101). In gastric cancer, the DNA methylation inhibitor 5-azacitidine (5-Aza) relieves the repression of the LATS2 promoter, thereby restoring Hippo pathway activity and reversing PAX6-mediated resistance to palbociclib (63). In addition, because NF-κB signaling contributes to therapeutic adaptation, the IκB kinase β inhibitor Bay 11–7082 has been shown to enhance the activity of palbociclib in liver cancer (109). Moreover, the cIAP1/2 antagonist LCL161 represents a rational partner for palbociclib, LCL161, a SMAC mimetic/IAP antagonist, promotes proteasomal degradation of cIAP1/2 and can shift palbociclib-induced cytostatic G1 arrest toward apoptotic cell death (68).

### 4.3 Targeting gene regulation and DNA damage repair networks

Aside from direct kinase inhibition, targeting the molecular machinery governing transcription and translation represents a promising therapeutic approach. In gastric cancer, the eIF4E inhibitor ribavirin restores sensitivity to abemaciclib by repressing cap-dependent translation of oncogenic mRNAs (103). BRD4, a member of the BET (bromodomain and extra-terminal) family of proteins, functions as an epigenetic regulator by binding to acetylated histones and promoting transcription of oncogenic genes. Inhibiting BRD4 with compounds such as JQ1 disrupts these transcriptional programs, thereby reducing expression of genes that drive tumor growth. When combined with the CDK4/6 inhibitor abemaciclib in gastric tumors, JQ1 exerts a synergistic effect by simultaneously blocking cell cycle progression and oncogenic transcription, enhancing antitumor efficacy (106). The nuclear export inhibitor selinexor (KPT-330) enhances RB-dependent repression of E2F by blocking XPO1-mediated RB nuclear export (112). In pancreatic cancer, disrupting the interaction between the RNA-binding protein HuR and *CCND1* mRNA using inhibitors such as CMLD-2 or pyrvinium pamoate reduces transcript stability and inhibits proliferation (62). In colorectal cancer models, the combination of PARP inhibitors and palbociclib enhanced anti-tumor immunity and decreased tumor burden (98). Furthermore, targeting the HSP90–CDC37 chaperone axis in colorectal cancer (CDC37 inhibitor DDO-6079) impairs the maturation of multiple oncogenic kinases, thereby reversing resistance to palbociclib (135).

### 4.4 Synergy with immunotherapy

CDK4/6 inhibitors are increasingly recognized for their role in modulating the tumor microenvironment. Palbociclib combined with PD-L1 or PD-1 antibodies improves antitumor immunity in pancreatic cancer (19). In colorectal cancer, the P-PBA/PLL nanocarrier combined with palbociclib enhances CD8+ T-cell activation (139), and the CD73 inhibitor AB680 further alleviates immune suppression by limiting extracellular adenosine production (137). These combinations demonstrate the potential of integrating CDK4/6 inhibitors with immunotherapy to enhance antitumor efficacy.

### 4.5 Metabolic targeting and chemotherapeutic sensitization

Metabolic vulnerabilities can potentiate CDK4/6 inhibitor activity. Glutaminase inhibition with CB-839 under glutamine-limiting conditions amplifies apoptosis, particularly in FBXO4-deficient tumor cells (89). Abemaciclib has been found to restore chemosensitivity by competitively inhibiting the efflux transporters ABCB1 and ABCG2 (141). Palbociclib enhances the efficacy of chemotherapeutics such as irinotecan by reversing RB phosphorylation and reducing HIF1α levels, while simultaneously mitigating systemic toxicity (138). These approaches highlight how metabolic and chemotherapeutic strategies can be integrated with CDK4/6 blockade to overcome intrinsic and acquired resistance.

In clinical trials of CDK4/6 inhibitors for GI cancers, combination regimens have been widely explored alongside single-agent therapy. Regarding combination applications, multiple dual-drug regimens have been investigated in different gastrointestinal tumor types. In pancreatic cancer, palbociclib was used in combination with ulixertinib (trial ongoing) (116), trametinib (119), and nab-paclitaxel (125). For metastatic colorectal cancer (with wild-type *KRAS*/*NRAS*/*BRAF*), palbociclib was combined with cetuximab (133). The safety profile of CDK4/6 inhibitor-based regimens in gastrointestinal cancers was broadly consistent with known class effects, with hematologic toxicity being the dominant adverse-event pattern. Neutropenia or decreased neutrophil count was the most frequently observed toxicity, but its incidence varied across studies depending on the regimen and reporting criteria. Other hematologic events included anemia, thrombocytopenia, leukopenia and lymphopenia, whereas non-hematologic toxicities included fatigue or asthenia, nausea, mucositis, rash, infusion-related reactions and gastrointestinal symptoms. QT prolongation should be interpreted as agent- or regimen-specific, particularly in studies involving ribociclib (**Table 2**).

**Table 2:** CDK4/6 inhibitor clinical trials in GI cancers.

Trial ID (NCT)	Drug	Cancer type	Phase	Key results	Ref
NCT03454035	Palbociclib + Ulixertinib	Pancreatic cancer	I	Recruiting	(116)
NCT06307249	Palbociclib + Bevacizumab	Colorectal cancer	I	Recruiting	*
NCT01684215	Palbociclib	n=4 (rectal 2, esophageal 1, pancreatic 1)	I	1 esophageal and 1 rectal cancer with SD ≥24 weeks. Neutropenia	(142)
NCT02478931	Palbociclib + Trametinib	n=8 (pancreatic 6, colorectal 1, GIST 1)	I	ORR: 22%, clinical benefit 56%, durable PFS up to 17.5+ months, well tolerated	(119)
NCT02501902	Palbociclib + Nab-paclitaxel	Pancreatic cancer (n=76)	Ib	ORR: 13.0%, mPFS: 5.3 months, mOS: 12.1 months. Neutropenia (76.3%), fatigue (52.6%), nausea (42.1%), anemia (40.8%)	(125)
NCT01898845	Ribociclib	Esophageal cancer (n=9)	I	ORR: 0%, DCR: 11.1%. Neutropenia (76%), QT prolongation (12%)	(143)
NCT02985125	Ribociclib + Everolimus	Metastatic pancreatic adenocarcinoma (n=12)	I	SD: 17%, mPFS: 1.8 months, mOS: 3.7 months. Neutropenia, thrombocytopenia, anemia	(121)
NCT03480256	Pyrotinib + SHR6390	Gastric Cancer(n=5)	I	ORR: 60%, DCR: 80%. Neutropenia	(104)
NCT03891784	Abemaciclib	Gastroenteropancreatic neuroendocrine tumors (n=21)	II	Active, not recruiting	*
NCT05358249	JDQ443 + Ribociclib	*KRAS* G12C mutant colorectal cancer	Ib/II	Active, not recruiting	*
NCT05865132	Palbociclib + Afatinib	Esophageal cancer (n=30)	II	No results posted yet	*
NCT03981614	Palbociclib + Binimetinib	*KRAS/NRAS* mutant colorectal cancer (n=42)	II	mPFS: 2.3 months, mOS: 7.7 months, ORR: 0%. Anemia (40%), neutropenia (35.7%).	(131)
NCT03446157	Palbociclib + Cetuximab	Metastatic colorectal cancer (*KRAS/NRAS/BRAF*) (n=12)	II	4-month DCR: 41.7%, mPFS: 5.3 months, mOS: 13.9 months. Lymphopenia (33%), neutropenia (17%), infusion reactions (42%), fatigue (25%).	(133)

ORR, objective response rate; SD, stable disease; DCR, disease control rate; mPFS, median progression-free survival; mOS, median overall survival.

*ClinicalTrials.gov; ongoing or recruiting trials with no published full-text articles available.

## 5 Conclusion and perspectives

CDK4/6 inhibitors exert dual antitumor effects in GI cancers by directly suppressing tumor cell proliferation and remodeling the tumor immune microenvironment. On the one hand, CDK4/6 inhibitors induce G1 phase arrest by inhibiting the canonical Cyclin D1-CDK4/6-RB signaling axis, thereby restraining sustained tumor cell proliferation. On the other hand, they can also promote the conversion of immunologically “cold” tumors into “hot” tumors through multiple mechanisms, including enhancement of antigen presentation, regulation of PD-L1 expression, suppression of immunosuppressive cell subsets, promotion of effector T-cell infiltration, and induction of SASP. However, despite their promising therapeutic potential in GI cancers, the clinical translation of CDK4/6 inhibitors still faces substantial challenges. GI cancers are characterized by marked molecular heterogeneity and complex signaling networks. In addition to abnormalities in the canonical RB pathway, aberrant activation of cooperating pathways, including PI3K/AKT/mTOR, RAS/RAF/MEK/ERK, Hippo, and NF-κB, can also profoundly influence tumor sensitivity to CDK4/6 inhibitors and contribute to both primary and acquired resistance.

Currently, no consensus has been reached on unified criteria for RB functional assessment, leading to heterogeneity in patient selection across relevant studies. In addition, some relevant biomarkers have not yet received FDA approval as companion diagnostics for gastrointestinal tumors. These markers remain largely exploratory, necessitating standardized detection protocols and prospective validation prior to clinical use. Therefore, the complexity of resistance mechanisms and the lack of clearly defined beneficiary populations remain major barriers to the further clinical application of CDK4/6 inhibitors (**Table 3**). Against this background, the development of mechanism-based combination strategies centered on CDK4/6 inhibitors has emerged as an important future direction. This complexity is compounded by the practical difficulties of toxicity management in combination settings, specifically the overlapping myelosuppression frequently observed when CDK4/6 inhibitors are integrated with cytotoxic chemotherapy. In the future, the clinical value of CDK4/6 inhibitors will likely be more fully realized in combination treatment paradigms based on mechanistic complementarity and resistance reversal. Looking forward, several critical questions must be addressed to advance the clinical application of CDK4/6 inhibitors in GI cancers. To overcome these barriers, future research must prioritize the development of a systematic and dynamic biomarker framework, moving beyond static biopsies toward ctDNA-based monitoring systems capable of tracking the clonal evolution of *RB1* mutations and other resistance-associated alterations in real-time. In parallel, future combination strategies should be tailored to tumor-specific biological contexts. In hepatocellular carcinoma, for example, a theoretical rationale exists for triple therapy combining a CDK4/6 inhibitor, an immune checkpoint inhibitor and an anti-angiogenic agent. CDK4/6 inhibition may enhance tumor immunogenicity and modulate T-cell activity, while anti-angiogenic therapy can potentiate immune checkpoint blockade through vascular normalization and attenuation of VEGF-mediated immunosuppression (92). Another important direction is the selective modulation of therapy-induced senescence. SASP has dual properties: in a specific molecular environment, it may exert an anti-tumor effect, while in other cases, it may promote tumor progression. The recruitment of macrophages, natural killer cells, neutrophils, and T cells mediated by SASP is considered to help limit tumorigenesis and eliminate apoptotic cells and cellular debris to initiate tissue remodeling. However, SASP may also directly affect immune cells, leading to immune dysfunction. It can not only activate macrophages to amplify inflammation, but also stimulate the generation of myeloid-derived immunosuppressive cells in the bone marrow and spleen, thereby inhibiting different components of innate immunity and adaptive immunity (144). The development of selective SASP modulators may provide a refined strategy to dissect the dual nature of SASP in the chronically inflamed microenvironment of gastrointestinal cancers. Specifically, the deleterious, pro-tumorigenic SASP components include IL-6, IL-8, and TNF-α, which fuel chronic inflammation, immunosuppression, and metastasis (145). Conversely, the protective, anti-tumorigenic SASP factors comprise IFN-γ, CXCL9/10 and IL-12, which enhance immune surveillance and support anti-tumor immunity (146–148). Therefore, these agents suppress deleterious pro-tumorigenic SASP components that drive persistent inflammation, immune suppression, and tumor progression, while preserving beneficial immune-regulatory SASP effects. This selective modulation offers a more nuanced therapeutic approach than global senolytic or senomorphic interventions. For instance, In colorectal cancer models, MM129 acts as an IL-6 inhibitor inhibits senescence-associated responses, thereby attenuating survival signals (149). Conversely, activation of tumor-suppressive SASP factors boosts antitumor immunity. EZH2 inhibition in pancreatic ductal adenocarcinoma elevates SASP-related chemokines CXCL9 and CXCL10, facilitating immune-mediated tumor elimination (148). Selective SASP inhibitors may be further combined with CDK4/6 inhibitors in the future for the treatment of GI cancers. Ultimately, the transition of CDK4/6 inhibitors from simple cell cycle stabilizers to sophisticated immune-metabolic modulators, supported by rationally designed, biomarker-enriched clinical trials that address these critical analytical gaps, will be essential for achieving meaningful clinical benefits in patients with GI cancers.

**Table 3:** Analytical validation status of biomarkers related to CDK4/6 inhibitor sensitivity and combination-therapy targets in GI cancers.

Biomarker category	Assay type	Representative markers	Analytical validation status
CDK4/6–RB axis protein markers	IHC	RB, p16, p21, p27, Cyclin D1, p53	Research-only/not FDA-approved CDx
CDK4/6–RB axis copy-number alterations	FISH	*RB1* deletion, *CDKN2A* loss, *CCND1* amplification	Research-only/not FDA-approved CDx
CDK4/6–RB axis genomic alterations	ctDNA NGS	*RB1, CDKN2A, CCND1, CDK4, CDK6*	Research-only/not FDA-approved CDx
PI3K/AKT/mTOR pathway activation markers	IHC	p-AKT, p-mTOR, p-4EBP1	Research-only/not FDA-approved CDx
PI3K/AKT/mTOR genomic alterations	ctDNA NGS	*PIK3CA, AKT1, MTOR*	Research-only/not FDA-approved CDx
Hippo pathway activity markers	IHC	YAP, TAZ	Research-only/not FDA-approved CDx
NF-κB pathway activity markers	IHC	p65/RelA, IκBα	Research-only/not FDA-approved CDx
NOTCH pathway markers	IHC	NOTCH1–4, JAG1, DLL family ligands, HES1, HEY1	Research-only/not FDA-approved CDx
*KRAS*/*NRAS* alterations	PCR ctDNA NGS	*KRAS*/*NRAS* status	FDA-approved CDx in selected colorectal cancer indications
PD-L1 expression	IHC	PD-L1	FDA-approved CDx in selected gastric, gastroesophageal junction, or esophageal cancer indications
*BRAF* V600E alteration	PCR ctDNA NGS	*BRAF* V600E mutation	FDA-approved CDx in selected metastatic colorectal cancer indications

CDx, companion diagnostic.

Source: U.S. Food and Drug Administration. List of Cleared or Approved Companion Diagnostic Devices (*In Vitro* and Imaging Tools). Available at: https://www.fda.gov/medical-devices/*in-vitro*-diagnostics/list-cleared-or-approved-companion-diagnostic-devices-*in-vitro*-and-imaging-tools.

## References

[1] Hanahan D . Hallmarks of cancer: New dimensions. Cancer Discov. (2022) 12:31–46. doi: 10.1158/2159-8290.CD-21-1059. PMID: 35022204

[2] Goel S Bergholz JS Zhao JJ . Targeting cdk4 and cdk6 in cancer. Nat Rev Cancer. (2022) 22:356–72. doi: 10.1038/s41568-022-00456-3. PMID: 35304604 PMC9149100

[3] Otto T Sicinski P . Cell cycle proteins as promising targets in cancer therapy. Nat Rev Cancer. (2017) 17:93–115. doi: 10.1038/nrc.2016.138. PMID: 28127048 PMC5345933

[4] Asghar U Witkiewicz AK Turner NC Knudsen ES . The history and future of targeting cyclin-dependent kinases in cancer therapy. Nat Rev Drug Discov. (2015) 14:130–46. doi: 10.1038/nrd4504. PMID: 25633797 PMC4480421

[5] Asciolla JJ Wu X Adamopoulos C Gavathiotis E Poulikakos PI . Resistance mechanisms and therapeutic strategies of cdk4 and cdk6 kina se targeting in cancer. Nat Cancer. (2025) 6:24–40. doi: 10.1038/s43018-024-00893-z. PMID: 39885369 PMC12207747

[6] Sherr CJ . Cancer cell cycles. Sci (New York NY). (1996) 274:1672–7. doi: 10.1126/science.274.5293.1672. PMID: 8939849

[7] Morrison L Loibl S Turner NC . The cdk4/6 inhibitor revolution - a game-changing era for breast cance r treatment. Nat Rev Clin Oncol. (2024) 21:89–105. doi: 10.1038/s41571-023-00840-4. PMID: 38082107

[8] Johnston SRD Harbeck N Hegg R Toi M Martin M Shao ZM . Abemaciclib combined with endocrine therapy for the adjuvant treatment of hr+, her2-, node-positive, high-risk, early breast cancer (monarch e). J Clin Oncol. (2020) 38:3987–98. doi: 10.1200/JCO.20.02514. PMID: 32954927 PMC7768339

[9] Slamon D Lipatov O Nowecki Z McAndrew N Kukielka-Budny B Stroyakovskiy D . Ribociclib plus endocrine therapy in early breast cancer. N Engl J Med. (2024) 390:1080–91. doi: 10.1056/NEJMoa2305488. PMID: 38507751

[10] Kalinsky K Bianchini G Hamilton E Graff SL Park KH Jeselsohn R . Abemaciclib plus fulvestrant in advanced breast cancer after progressi on on cdk4/6 inhibition: Results from the phase iii postmonarch trial. J Clin Oncol. (2025) 43:1101–12. doi: 10.1200/JCO-24-02086. PMID: 39693591 PMC11936477

[11] Voutsadakis IA . Development of cdk4/6 inhibitors in gastrointestinal cancers: Biomarke rs to move forward. Curr Issues Mol Biol. (2025) 47:454. doi: 10.3390/cimb47060454. PMID: 40699853 PMC12192106

[12] Zhan T Betge J Schulte N Dreikhausen L Hirth M Li M . Digestive cancers: Mechanisms, therapeutics and management. Signal Transduction Targeted Ther. (2025) 10:24. doi: 10.1038/s41392-024-02097-4. PMID: 39809756 PMC11733248

[13] Bray F Laversanne M Sung H Ferlay J Siegel RL Soerjomataram I . Global cancer statistics 2022: Globocan estimates of incidence and mor tality worldwide for 36 cancers in 185 countries. CA: A Cancer J For Clin. (2024) 74:229–63. doi: 10.3322/caac.21834. PMID: 38572751

[14] Antonarelli G Taurelli Salimbeni B Marra A Esposito A Locatelli MA Trapani D . The cdk4/6 inhibitors biomarker landscape: The most relevant biomarker s of response or resistance for further research and potential clinica l utility. Crit Rev Oncol Hematol. (2023) 192:104148. doi: 10.1016/j.critrevonc.2023.104148. PMID: 37783318

[15] Zhang S Xu Q Sun W Zhou J Zhou J . Immunomodulatory effects of cdk4/6 inhibitors. Biochim Biophys Acta Rev Cancer. (2023) 1878:188912. doi: 10.1016/j.bbcan.2023.188912. PMID: 37182667

[16] Venkadakrishnan VB Yamada Y Weng K Idahor O Beltran H . Significance of rb loss in unlocking phenotypic plasticity in advanced cancers. Mol Cancer Res MCR. (2023) 21:497–510. doi: 10.1158/1541-7786.MCR-23-0045. PMID: 37052520 PMC10239360

[17] Malumbres M Barbacid M . Cell cycle, cdks and cancer: A changing paradigm. Nat Rev Cancer. (2009) 9:153–66. doi: 10.1038/nrc2602. PMID: 19238148

[18] Thangavelu PU Lin C-Y Forouz F Tanaka K Dray E Duijf PHG . The rb protein: More than a sentry of cell cycle entry. Trends Mol Med. (2025) 31:1124–39. doi: 10.1016/j.molmed.2025.04.001. PMID: 40300971

[19] Knudsen ES Kumarasamy V Chung S Ruiz A Vail P Tzetzo S . Targeting dual signalling pathways in concert with immune checkpoints for the treatment of pancreatic cancer. Gut. (2021) 70:127–38. doi: 10.1136/gutjnl-2020-321000. PMID: 32424005 PMC7671951

[20] Schneider JS Khaled NB Ye L Wimmer R Hammann L Weich A . Efficacy of cdk4/6 inhibition in colorectal cancer and the role of p16 expression in predicting drug resistance. Cell Oncol (Dordrecht Netherlands). (2025) 48:1363–75. doi: 10.1007/s13402-025-01080-7. PMID: 40522623 PMC12528337

[21] Bury M Le Calvé B Ferbeyre G Blank V Lessard F . New insights into cdk regulators: Novel opportunities for cancer thera py. Trends Cell Biol. (2021) 31:331–44. doi: 10.1016/j.tcb.2021.01.010. PMID: 33676803

[22] Abedizadeh R Majidi F Khorasani HR Abedi H Sabour D . Colorectal cancer: A comprehensive review of carcinogenesis, diagnosis , and novel strategies for classified treatments. Cancer Metastasis Rev. (2024) 43:729–53. doi: 10.1007/s10555-023-10158-3. PMID: 38112903

[23] Kamisawa T Wood LD Itoi T Takaori K . Pancreatic cancer. Lancet (London England). (2016) 388:73–85. doi: 10.1016/S0140-6736(16)00141-0. PMID: 26830752

[24] Lin J Cao Y Yang X Li G Shi Y Wang D . Mutational spectrum and precision oncology for biliary tract carcinoma. Theranostics. (2021) 11:4585–98. doi: 10.7150/thno.56539. PMID: 33754015 PMC7978308

[25] Bae HJ Kang SK Kwon WS Jeong I Park S Kim TS . P16 methylation is a potential predictive marker for abemaciclib sensi tivity in gastric cancer. Biochem Pharmacol. (2021) 183:114320. doi: 10.1016/j.bcp.2020.114320. PMID: 33161023

[26] Jiang D Zhang X Chen W Peng R Zhang X Gao F . Abnormal p16 expression and prognostic significance in esophageal squa mous cell carcinoma. Histol Histopathol. (2024) 39:201–9. Available online at: https://pubmed.ncbi.nlm.nih.gov/37132443/. 10.14670/HH-18-619 37132443

[27] Li P Zhang X Gu L Zhou J Deng D . P16 methylation increases the sensitivity of cancer cells to the cdk4/ 6 inhibitor palbociclib. PLoS One. (2019) 14:e0223084. doi: 10.1371/journal.pone.0223084. PMID: 31652270 PMC6814222

[28] Razavipour SF Harikumar KB Slingerland JM . P27 as a transcriptional regulator: New roles in development and cance r. Cancer Res. (2020) 80:3451–8. doi: 10.1158/0008-5472.CAN-19-3663. PMID: 32341036

[29] Rahmani F Zandigohar M Safavi P Behzadi M Ghorbani Z Payazdan M . The interplay between noncoding rnas and p21 signaling in gastrointest inal cancer: From tumorigenesis to metastasis. Curr Pharm Des. (2023) 29:766–76. doi: 10.2174/1381612829666230306123455. PMID: 36876835

[30] Rampioni Vinciguerra GL Dall'Acqua A Segatto I Mattevi MC Russo F Favero A . P27kip1 expression and phosphorylation dictate palbociclib sensitivity in kras-mutated colorectal cancer. Cell Death Dis. (2021) 12:951. doi: 10.1038/s41419-021-04241-2. PMID: 34654798 PMC8519959

[31] Wang L Han H Dong L Wang Z Qin Y . Function of p21 and its therapeutic effects in esophageal cancer. Oncol Lett. (2021) 21:136. doi: 10.3892/ol.2020.12397. PMID: 33552255 PMC7798030

[32] Yu M Wu W Sun Y Yan H Zhang L Wang Z . Frmd8 targets both cdk4 activation and rb degradation to suppress colo n cancer growth. Cell Rep. (2023) 42:112886. doi: 10.1016/j.celrep.2023.112886. PMID: 37527040

[33] Wang J Xiu J Farrell A Baca Y Arai H Battaglin F . Mutational analysis of microsatellite-stable gastrointestinal cancer w ith high tumour mutational burden: A retrospective cohort study. Lancet Oncol. (2023) 24:151–61. doi: 10.1016/S1470-2045(22)00783-5. PMID: 36681091 PMC10599647

[34] Sittithumcharee G Suppramote O Vaeteewoottacharn K Sirisuksakun C Jamnongsong S Laphanuwat P . Dependency of cholangiocarcinoma on cyclin d-dependent kinase activity. Hepatol (Baltimore Md). (2019) 70:1614–30. doi: 10.1002/hep.30704. PMID: 31077409

[35] Wang J Li Q Yuan J Wang J Chen Z Liu Z . Cdk4/6 inhibitor-shr6390 exerts potent antitumor activity in esophagea l squamous cell carcinoma by inhibiting phosphorylated rb and inducing g1 cell cycle arrest. J Transl Med. (2017) 15:127. doi: 10.1186/s12967-017-1231-7. PMID: 28578693 PMC5457542

[36] Cui Y Li H Zhan H Han T Dong Y Tian C . Identification of potential biomarkers for liver cancer through gene m utation and clinical characteristics. Front Oncol. (2021) 11:733478. doi: 10.3389/fonc.2021.733478. PMID: 34604069 PMC8484954

[37] Fang M Wu H-K Pei Y Zhang Y Gao X He Y . E3 ligase mg53 suppresses tumor growth by degrading cyclin d1. Signal Transduction Targeted Ther. (2023) 8:263. doi: 10.1038/s41392-023-01458-9. PMID: 37414783 PMC10326024

[38] Lian Z Lee EK Bass AJ Wong KK Klein-Szanto AJ Rustgi AK . Fbxo4 loss facilitates carcinogen induced papilloma development in mic e. Cancer Biol Ther. (2015) 16:750–5. doi: 10.1080/15384047.2015.1026512. PMID: 25801820 PMC4622573

[39] Freeman-Cook K Hoffman RL Miller N Almaden J Chionis J Zhang Q . Expanding control of the tumor cell cycle with a cdk2/4/6 inhibitor. Cancer Cell. (2021) 39:1404–1421.e11. doi: 10.1016/j.ccell.2021.08.009. PMID: 34520734

[40] Min A Kim JE Kim Y-J Lim JM Kim S Kim JW . Cyclin e overexpression confers resistance to the cdk4/6 specific inhi bitor palbociclib in gastric cancer cells. Cancer Lett. (2018) 430:123–32. doi: 10.1016/j.canlet.2018.04.037. PMID: 29729292

[41] Ding X He M Chan AWH Song QX Sze SC Chen H . Genomic and epigenomic features of primary and recurrent hepatocellula r carcinomas. Gastroenterology. (2020) 157(6):1630-45.e6. S0016–5085(20)30659-4. doi: 10.1053/j.gastro.2019.09.056. PMID: 31560893

[42] Wang H Liao P Zeng SX Lu H . Co-targeting p53-r249s and cdk4 synergistically suppresses survival of hepatocellular carcinoma cells. Cancer Biol Ther. (2020) 21:269–77. doi: 10.1080/15384047.2019.1685289. PMID: 31747859 PMC7012101

[43] Urbach L Wieland L Penz F Samuel RD Küffer S Klein L . Tp53missense-specific transcriptional plasticity drives resist ance against cell cycle inhibitors in pancreatic cancer. Sci Adv. (2025) 11:eadu2339. doi: 10.1126/sciadv.adu2339. PMID: 40614202 PMC12227071

[44] Liu Y Li Z Zhang J Liu W Guan S Zhan Y . Dynll1 accelerates cell cycle via ilf2/cdk4 axis to promote hepatocell ular carcinoma development and palbociclib sensitivity. Br J Cancer. (2024) 131:243–57. doi: 10.1038/s41416-024-02719-2. PMID: 38824222 PMC11263598

[45] Hsieh F-S Chen Y-L Hung M-H Chu P-Y Tsai M-H Chen L-J . Palbociclib induces activation of ampk and inhibits hepatocellular car cinoma in a cdk4/6-independent manner. Mol Oncol. (2017) 11:1035–49. doi: 10.1002/1878-0261.12072. PMID: 28453226 PMC5537702

[46] Price P Ganugapati U Gatalica Z Kakadekar A Macpherson J Quenneville L . Reinventing nuclear histo-score utilizing inherent morphologic cutoffs : Blue-brown color h-score (bbc-hs). Appl Immunohistochem Mol Morphol. (2023) 31:500–6. doi: 10.1097/PAI.0000000000001095. PMID: 36625446 PMC10396076

[47] Goldsmith JD Troxell ML Roy-Chowdhuri S Colasacco CF Edgerton ME Fitzgibbons PL . Principles of analytic validation of immunohistochemical assays: Guide line update. Arch Pathol Lab Med. (2024) 148:e111–53. doi: 10.5858/arpa.2023-0483-CP. PMID: 38391878

[48] Jennings LJ Arcila ME Corless C Kamel-Reid S Lubin IM Pfeifer J . Guidelines for validation of next-generation sequencing-based oncology panels: A joint consensus recommendation of the association for molec ular pathology and college of american pathologists. J Mol Diagn. (2017) 19:341–65. doi: 10.1016/j.jmoldx.2017.01.011. PMID: 28341590 PMC6941185

[49] Pascual J Attard G Bidard FC Curigliano G De Mattos-Arruda L Diehn M . Esmo recommendations on the use of circulating tumour dna assays for p atients with cancer: A report from the esmo precision medicine working group. Ann Oncol. (2022) 33:750–68. doi: 10.1016/j.annonc.2022.05.520. PMID: 35809752

[50] Kang GH Lee HJ Hwang KS Lee S Kim J-H Kim J-S . Aberrant cpg island hypermethylation of chronic gastritis, in relation to aging, gender, intestinal metaplasia, and chronic inflammation. Am J Pathol. (2003) 163:1551–6. doi: 10.1016/S0002-9440(10)63511-0. PMID: 14507661 PMC1868290

[51] Zhu G Pei L Xia H Tang Q Bi F . Role of oncogenic Kras in the prognosis, diagnosis and treatment of co lorectal cancer. Mol Cancer. (2021) 20:143. doi: 10.1186/s12943-021-01441-4. PMID: 34742312 PMC8571891

[52] Singhal A Li BT O'Reilly EM . Targeting Kras in cancer. Nat Med. (2024) 30:969–83. doi: 10.1038/s41591-024-02903-0. PMID: 38637634 PMC11845254

[53] Blangé D Stroes CI Derks S Bijlsma MF van Laarhoven HWM . Resistance mechanisms to Her2-targeted therapy in gastroesophageal ade nocarcinoma: a systematic review. Cancer Treat Rev. (2022) 108:102418. doi: 10.1016/j.ctrv.2022.102418. PMID: 35689885

[54] Hung Y-H Hsu S-H Hou Y-C Chu P-Y Su Y-Y Shan Y-S . Semaphorin 6c suppresses proliferation of pancreatic cancer cells via inhibition of the Akt/Gsk3/B-catenin/cyclin D1 pathway. Int J Mol Sci. (2022) 23:2608. doi: 10.3390/ijms23052608. PMID: 35269749 PMC8910270

[55] Qian Y Wu X Wang H Hou G Han X Song W . Pak1 silencing is synthetic lethal with Cdk4/6 inhibition in gastric c ancer cells via regulating Pdk1 expression. Hum Cell. (2020) 33:377–85. doi: 10.1007/s13577-019-00317-6. PMID: 31919718

[56] Cao L Weng K Li L Lin G Zhao Y Gao Y . Batf2 inhibits the stem cell-like properties and chemoresistance of ga stric cancer cells through Pten/Akt/B-catenin pathway. Theranostics. (2024) 14:7007–22. doi: 10.7150/thno.98389. PMID: 39629124 PMC11610130

[57] Lim SH Lee S-Y Hong JY Lee J Kim ST . Cdk4/6 inhibition to resensitize Braf/Egfr inhibitor in patient-derive d Braf/Pten-mutant colon cancer cells. Trans Cancer Res. (2024) 13:3695–703. doi: 10.21037/tcr-24-20. PMID: 39145064 PMC11319972

[58] Callegari E Guerriero P Bassi C D'Abundo L Frassoldati A Simoni E . Mir-199a-3p increases the anti-tumor activity of palbociclib in liver cancer models. Mol Ther Nucleic Acids. (2022) 29:538–49. doi: 10.1016/j.omtn.2022.07.015. PMID: 36035756 PMC9395755

[59] Im WR Lee H-S Lee Y-S Lee J-S Jang H-J Kim S-Y . A regulatory noncoding Rna, Nc886, suppresses esophageal cancer by inh ibiting the Akt pathway and cell cycle progression. Cells. (2020) 9:801. doi: 10.3390/cells9040801. PMID: 32225025 PMC7226379

[60] Ma S Meng Z Chen R Guan K-L . The Hippo pathway: Biology and pathophysiology. Annu Rev Biochem. (2019) 88:577–604. doi: 10.1146/annurev-biochem-013118-111829. PMID: 30566373

[61] Ou W-B Ni N Zuo R Zhuang W Zhu M Kyriazoglou A . Cyclin D1 is a mediator of gastrointestinal stromal tumor kit-independ ence. Oncogene. (2019) 38:6615–29. doi: 10.1038/s41388-019-0894-3. PMID: 31371779

[62] Dhir T Schultz CW Jain A Brown SZ Haber A Goetz A . Abemaciclib is effective against pancreatic cancer cells and synergize s with Hur and Yap1 inhibition. Mol Cancer Res MCR. (2019) 17:2029–41. doi: 10.1158/1541-7786.MCR-19-0589. PMID: 31383722 PMC6794000

[63] Zhang Y He L-J Huang L-L Yao S Lin N Li P . Oncogenic Pax6 elicits Cdk4/6 inhibitor resistance by epigenetically i nactivating the Lats2-Hippo signaling pathway. Clin Transl Med. (2021) 11:e503. doi: 10.1002/ctm2.503. PMID: 34459131 PMC8382979

[64] Wen Y Yang X Li S Huang L Chen J Tan L . Targeting Cdk4/6 suppresses colorectal cancer by destabilizing Yap1. MedComm. (2025) 6:e70103. doi: 10.1002/mco2.70103. PMID: 39968498 PMC11832431

[65] Merga YJ O'Hara A Burkitt MD Duckworth CA Probert CS Campbell BJ . Importance of the alternative Nf-Kb activation pathway in inflammation -associated gastrointestinal carcinogenesis. Am J Physiol Gastrointestinal Liver Physiol. (2016) 310:G1081–90. doi: 10.1152/ajpgi.00026.2016. PMID: 27102559

[66] Sokolova O Naumann M . Nf-Kb signaling in gastric cancer. Toxins. (2017) 9:119. doi: 10.3390/toxins9040119. PMID: 28350359 PMC5408193

[67] Lalaoui N Vaux DL . Recent advances in understanding inhibitor of apoptosis proteins. F1000Research. (2018) 7:F1000. doi: 10.12688/f1000research.16439.1. PMID: . Faculty Rev-889. 30631429 PMC6281012

[68] Menapree P Duangthim N Sae-Fung A Sonkaew S Jitkaew S . Cdk4/6 inhibitors upregulate Ciap1/2, and Smac mimetic Lcl161 enhances their antitumor effects in cholangiocarcinoma cells. Sci Rep. (2025) 15:6826. doi: 10.1038/s41598-025-90997-y. PMID: 40000765 PMC11861974

[69] Shi Q Xue C Zeng Y Yuan X Chu Q Jiang S . Notch signaling pathway in cancer: From mechanistic insights to target ed therapies. Signal Transduction Targeted Ther. (2024) 9:128. doi: 10.1038/s41392-024-01828-x. PMID: 38797752 PMC11128457

[70] Gaston K Tsitsilianos M-A Wadey K Jayaraman P-S . Misregulation of the proline rich homeodomain (Prh/Hhex) protein in ca ncer cells and its consequences for tumour growth and invasion. Cell Bioscience. (2016) 6:12. doi: 10.1186/s13578-016-0077-7. PMID: 26877867 PMC4752775

[71] Kitchen P Lee KY Clark D Lau N Lertsuwan J Sawasdichai A . A runaway Prh/Hhex-Notch3-positive feedback loop drives cholangiocarci noma and determines response to Cdk4/6 inhibition. Cancer Res. (2020) 80:757–70. doi: 10.1158/0008-5472.CAN-19-0942. PMID: 31843982

[72] Tasioudi KE Sakellariou S Levidou G Theodorou D Michalopoulos NV Patsouris E . Immunohistochemical and molecular analysis of Pi3k/Akt/Mtor pathway in esophageal carcinoma. APMIS. (2015) 123:639–47. doi: 10.1111/apm.12398. PMID: 25912437

[73] Zanconato F Cordenonsi M Piccolo S . Yap/Taz at the roots of cancer. Cancer Cell. (2016) 29:783–803. doi: 10.1016/j.ccell.2016.05.005. PMID: 27300434 PMC6186419

[74] Zhou F Wei H Ding A Qiu W Feng L Zhou Q . Different cellular localization of Nf-Kb p65 expression as an indicato r of different prognoses of stage I-iii gastric cancer patients. Clin Transl Sci. (2013) 6:381–5. doi: 10.1111/cts.12065. PMID: 24127926 PMC5350890

[75] Katoh M Katoh M . Precision medicine for human cancers with Notch signaling dysregulatio n (review). Int J Mol Med. (2020) 45:279–97. doi: 10.3892/ijmm.2019.4418. PMID: 31894255 PMC6984804

[76] Muñoz-Fontela C Mandinova A Aaronson SA Lee SW . Emerging roles of P53 and other tumour-suppressor genes in immune regu lation. Nat Rev Immunol. (2016) 16:741–50. doi: 10.1038/nri.2016.99. PMID: 27667712 PMC5325695

[77] Liu Y Li C Lu Y Liu C Yang W . Tumor microenvironment-mediated immune tolerance in development and tr eatment of gastric cancer. Front Immunol. (2022) 13:1016817. doi: 10.3389/fimmu.2022.1016817. PMID: 36341377 PMC9630479

[78] Negura I Pavel-Tanasa M Danciu M . Regulatory T cells in gastric cancer: Key controllers from pathogenesi s to therapy. Cancer Treat Rev. (2023) 120:102629. doi: 10.1016/j.ctrv.2023.102629. PMID: 37769435

[79] Kobayashi H Gieniec KA Lannagan TRM Wang T Asai N Mizutani Y . The origin and contribution of cancer-associated fibroblasts in colore ctal carcinogenesis. Gastroenterology. (2022) 162:890–906. doi: 10.1053/j.gastro.2021.11.037. PMID: 34883119 PMC8881386

[80] Goel S DeCristo MJ Watt AC BrinJones H Sceneay J Li BB . Cdk4/6 inhibition triggers anti-tumour immunity. Nature. (2017) 548:471–5. doi: 10.1038/nature23465. PMID: 28813415 PMC5570667

[81] DhatChinamoorthy K Colbert JD Rock KL . Cancer immune evasion through loss of Mhc class I antigen presentation. Front Immunol. (2021) 12:636568. doi: 10.3389/fimmu.2021.636568. PMID: 33767702 PMC7986854

[82] Yu J Qiu P Ai J Liu B Han G-Z Zhu F . Endogenous retrovirus activation: Potential for immunology and clinica l applications. Natl Sci Rev. (2024) 11:nwae034. doi: 10.1093/nsr/nwae034. PMID: 38495812 PMC10941811

[83] Lou L Deng T Yuan Q Wang L Wang Z Li X . Targeted silencing of Socs1 by Dnmt1 promotes stemness of human liver cancer stem-like cells. Cancer Cell Int. (2024) 24:206. doi: 10.1186/s12935-024-03322-4. PMID: 38867242 PMC11170857

[84] Wong KK . Dnmt1 as a therapeutic target in pancreatic cancer: Mechanisms and cli nical implications. Cell Oncol (Dordrecht Netherlands). (2020) 43:779–92. doi: 10.1007/s13402-020-00526-4. PMID: 32504382 PMC12990712

[85] Peng X-M Shi X-P Chen H Cao L-Y Zuo H-J Guo J-Q . Decitabine promotes degradation of Dnmt1 and Ezh2 via the ubiquitinati on pathway and inhibits colorectal cancer progression. Cell Oncol (Dordrecht Netherlands). (2025) 49:9. doi: 10.1007/s13402-025-01136-8. PMID: 41460614 PMC12748097

[86] Zhao H Ning S Nolley R Scicinski J Oronsky B Knox SJ . The immunomodulatory anticancer agent, Rrx-001, induces an interferon response through epigenetic induction of viral mimicry. Clin Epigenet. (2017) 9:4. doi: 10.1186/s13148-017-0312-z. PMID: 28149332 PMC5270305

[87] Roulois D Loo Yau H Singhania R Wang Y Danesh A Shen SY . DNA-demethylating agents target colorectal cancer cells by inducing vi ral mimicry by endogenous transcripts. Cell. (2015) 162:961–73. doi: 10.1016/j.cell.2015.07.056. PMID: 26317465 PMC4843502

[88] Bever KM Thomas DL Zhang J Diaz Rivera EA Rosner GL Zhu Q . A feasibility study of combined epigenetic and vaccine therapy in adva nced colorectal cancer with pharmacodynamic endpoint. Clin Epigenet. (2021) 13:25. doi: 10.1186/s13148-021-01014-8. PMID: 33531075 PMC7856736

[89] Schaer DA Beckmann RP Dempsey JA Huber L Forest A Amaladas N . The Cdk4/6 inhibitor abemaciclib induces a T cell inflamed tumor micro environment and enhances the efficacy of Pd-L1 checkpoint blockade. Cell Rep. (2018) 22:2978–94. doi: 10.1016/j.celrep.2018.02.053. PMID: 29539425

[90] He L Peng Y Leong L-L Zhou J Tang D Wang W . Cdk4/6 inhibition induces Cd8+ T cell antitumor immunity vi a Mif-induced functional orchestration of tumor-associated macrophages. Advanced Sci (Weinheim Baden-Wurttemberg Germany). (2025) e11330. doi: 10.1002/advs.202511330. PMID: 41082324 PMC13292170

[91] Deng J Wang ES Jenkins RW Li S Dries R Yates K . Cdk4/6 inhibition augments antitumor immunity by enhancing T-cell acti vation. Cancer Discov. (2018) 8:216–33. doi: 10.1158/2159-8290.CD-17-0915. PMID: 29101163 PMC5809273

[92] Cai X Yin G Chen S Tacke F Guillot A Liu H . Cdk4/6 inhibition enhances T-cell immunotherapy on hepatocellular carc inoma cells by rejuvenating immunogenicity. Cancer Cell Int. (2024) 24:215. doi: 10.1186/s12935-024-03351-z. PMID: 38902716 PMC11188513

[93] Zhang J Bu X Wang H Zhu Y Geng Y Nihira NT . Cyclin D-Cdk4 kinase destabilizes Pd-L1 via Cullin 3-Spop to control c ancer immune surveillance. Nature. (2018) 553:91–5. doi: 10.1038/nature25015. PMID: 29160310 PMC5754234

[94] Jin X Ding D Yan Y Li H Wang B Ma L . Phosphorylated Rb promotes cancer immunity by inhibiting Nf-Kb activat ion and Pd-L1 expression. Mol Cell. (2019) 73:22–35.e6. doi: 10.1016/j.molcel.2018.10.034. PMID: 30527665 PMC8968458

[95] Zhou L Ma B Ruscetti M . Cellular senescence offers distinct immunological vulnerabilities in c ancer. Trends Cancer. (2025) 11:334–50. doi: 10.1016/j.trecan.2024.11.010. PMID: 39732594 PMC11981858

[96] Ruscetti M Morris J Mezzadra R Russell J Leibold J Romesser PB . Senescence-induced vascular remodeling creates therapeutic vulnerabili ties in pancreas cancer. Cell. (2020) 181:424–441.e21. doi: 10.1016/j.cell.2020.03.008. PMID: 32234521 PMC7278897

[97] Wong PY Chan CYK Xue HDG Goh CC Cheu JWS Tse APW . Cell cycle inhibitors activate the hypoxia-induced Ddx41/Sting pathway to mediate antitumor immune response in liver cancer. JCI Insight. (2024) 9:e170532. doi: 10.1172/jci.insight.170532. PMID: 39388278 PMC11601891

[98] Wang T Liu W Shen Q Tao R Li C Shen Q . Combination of Parp inhibitor and Cdk4/6 inhibitor modulates Cgas/Stin g-dependent therapy-induced senescence and provides "one-two punch" opportunity with anti-Pd-L1 therapy in colorectal cancer. Cancer Sci. (2023) 114:4184–201. doi: 10.1111/cas.15961. PMID: 37702298 PMC10637067

[99] Zhang X Wang Y Zhang X Shen Y Yang K Ma Q . Intact regulation of G1/S transition renders esophageal squamous cell carcinoma sensitive to Pi3kα inhibitors. Signal Transduction Targeted Ther. (2023) 8:153. doi: 10.1038/s41392-023-01359-x. PMID: 37041169 PMC10090078

[100] Zhou J Wu Z Zhang Z Goss L McFarland J Nagaraja A . Pan-erbb kinase inhibition augments cdk4/6 inhibitor efficacy in oesop hageal squamous cell carcinoma. Gut. (2022) 71:665–75. doi: 10.1136/gutjnl-2020-323276. PMID: 33789967 PMC8921580

[101] Li F Xu Y Liu B Singh PK Zhao W Jin J . Yap1-mediated cdk6 activation confers radiation resistance in esophage al cancer - rationale for the combination of yap1 and cdk4/6 inhibitor s in esophageal cancer. Clin Cancer Res. (2019) 25:2264–77. doi: 10.1158/1078-0432.CCR-18-1029. PMID: 30563933

[102] Qie S Yoshida A Parnham S Oleinik N Beeson GC Beeson CC . Targeting glutamine-addiction and overcoming cdk4/6 inhibitor resistan ce in human esophageal squamous cell carcinoma. Nat Commun. (2019) 10:1296. doi: 10.1038/s41467-019-09179-w. PMID: 30899002 PMC6428878

[103] Zha H-L Chen W Shi W Liao Y-Y . Inhibition of eukaryotic initiating factor eif4e overcomes abemaciclib resistance in gastric cancer. Curr Med Sci. (2023) 43:927–34. doi: 10.1007/s11596-023-2789-3. PMID: 37752406

[104] Chen Z Xu Y Gong J Kou F Zhang M Tian T . Pyrotinib combined with cdk4/6 inhibitor in her2-positive metastatic g astric cancer: a promising strategy from avatar mouse to patients. Clin Transl Med. (2020) 10:e148. doi: 10.1002/ctm2.148. PMID: 32898333 PMC7424666

[105] Peng K Zhang F Wang Y Sahgal P Li T Zhou J . Development of combination strategies for focal adhesion kinase inhibi tion in diffuse gastric cancer. Clin Cancer Res. (2023) 29:197–208. doi: 10.1158/1078-0432.CCR-22-1609. PMID: 36278961 PMC9812865

[106] Feng M Xu H Zhou W Pan Y . The brd4 inhibitor jq1 augments the antitumor efficacy of abemaciclib in preclinical models of gastric carcinoma. J Exp Clin Cancer Res. (2023) 42:44. doi: 10.1186/s13046-023-02615-2. PMID: 36755269 PMC9909925

[107] Xu H Chen K Shang R Chen X Zhang Y Song X . Alpelisib combination treatment as novel targeted therapy against hepa tocellular carcinoma. Cell Death Dis. (2021) 12:920. doi: 10.1038/s41419-021-04206-5. PMID: 34625531 PMC8501067

[108] Prawira A Le TBU Vu TC Huynh H . Ribociclib enhances infigratinib-induced cancer cell differentiation a nd delays resistance in fgfr-driven hepatocellular carcinoma. Liver Int. (2021) 41:608–20. doi: 10.1111/liv.14728. PMID: 33179425 PMC7894323

[109] Sheng J Kohno S Okada N Okahashi N Teranishi K Matsuda F . Treatment of retinoblastoma 1-intact hepatocellular carcinoma with cyc lin-dependent kinase 4/6 inhibitor combination therapy. Hepatol (Baltimore Md). (2021) 74:1971–93. doi: 10.1002/hep.31872. PMID: 33931882

[110] Jo H Park Y Kim T Kim J Lee JS Kim SY . Modulation of sirt3 expression through cdk4/6 enhances the anti-cancer effect of sorafenib in hepatocellular carcinoma cells. BMC Cancer. (2020) 20:332. doi: 10.1186/s12885-020-06822-4. PMID: 32306906 PMC7168998

[111] Digiacomo G Fumarola C La Monica S Bonelli MA Cretella D Alfieri R . Simultaneous combination of the cdk4/6 inhibitor palbociclib with rego rafenib induces enhanced anti-tumor effects in hepatocarcinoma cell li nes. Front Oncol. (2020) 10:563249. doi: 10.3389/fonc.2020.563249. PMID: 33072590 PMC7539564

[112] Wang H Yuan S Zheng Q Zhang S Zhang Q Ji S . Dual inhibition of cdk4/6 and xpo1 induces senescence with acquired vu lnerability to crbn-based protac drugs. Gastroenterology. (2024) 166:1130–1144.e8. doi: 10.1053/j.gastro.2024.01.025. PMID: 38262581

[113] Song X Liu X Wang H Wang J Qiao Y Cigliano A . Combined cdk4/6 and pan-mtor inhibition is synergistic against intrahe patic cholangiocarcinoma. Clin Cancer Res. (2019) 25:403–13. doi: 10.1158/1078-0432.CCR-18-0284. PMID: 30084835 PMC6423983

[114] Song X Xu H Wang P Wang J Affo S Wang H . Focal adhesion kinase (fak) promotes cholangiocarcinoma development an d progression via yap activation. J Hepatol. (2021) 75:888–99. doi: 10.1016/j.jhep.2021.05.018. PMID: 34052254 PMC8453055

[115] Arora M Bogenberger JM Abdelrahman AM Yonkus J Alva-Ruiz R Leiting JL . Synergistic combination of cytotoxic chemotherapy and cyclin-dependent kinase 4/6 inhibitors in biliary tract cancers. Hepatol (Baltimore Md). (2022) 75:43–58. doi: 10.1002/hep.32102. PMID: 34407567

[116] Goodwin CM Waters AM Klomp JE Javaid S Bryant KL Stalnecker CA . Combination therapies with cdk4/6 inhibitors to treat kras-mutant panc reatic cancer. Cancer Res. (2023) 83:141–57. doi: 10.1158/0008-5472.CAN-22-0391. PMID: 36346366 PMC9812941

[117] Heilmann AM Perera RM Ecker V Nicolay BN Bardeesy N Benes CH . Cdk4/6 and igf1 receptor inhibitors synergize to suppress the growth o f p16ink4a-deficient pancreatic cancers. Cancer Res. (2014) 74:3947–58. doi: 10.1158/0008-5472.CAN-13-2923. PMID: 24986516 PMC4122288

[118] Maust JD Frankowski-McGregor CL Bankhead A Simeone DM Sebolt-Leopold JS . Cyclooxygenase-2 influences response to cotargeting of mek and cdk4/6 in a subpopulation of pancreatic cancers. Mol Cancer Ther. (2018) 17:2495–506. doi: 10.1158/1535-7163.MCT-18-0082. PMID: 30254182 PMC6279520

[119] Kato S Adashek JJ Shaya J Okamura R Jimenez RE Lee S . Concomitant mek and cyclin gene alterations: implications for response to targeted therapeutics. Clin Cancer Res. (2021) 27:2792–7. doi: 10.1158/1078-0432.CCR-20-3761. PMID: 33472910 PMC11005753

[120] Willobee BA Gaidarski AA Dosch AR Castellanos JA Dai X Mehra S . Combined blockade of mek and cdk4/6 pathways induces senescence to imp rove survival in pancreatic ductal adenocarcinoma. Mol Cancer Ther. (2021) 20:1246–56. doi: 10.1158/1535-7163.MCT-19-1043. PMID: 34001634 PMC8260447

[121] Weinberg BA Wang H Witkiewicz AK Marshall JL He AR Vail P . A phase i study of ribociclib plus everolimus in patients with metasta tic pancreatic adenocarcinoma refractory to chemotherapy. J Pancreat Cancer. (2020) 6:45–54. doi: 10.1089/pancan.2020.0005. PMID: 32642630 PMC7337242

[122] Tang H Ge Y You T Li X Wang Y Cheng Y . A real-world analysis of trametinib in combination with hydroxychloroq uine or cdk4/6 inhibitor as third- or later-line therapy in metastatic pancreatic adenocarcinoma. BMC Cancer. (2023) 23:958. doi: 10.1186/s12885-023-11464-3. PMID: 37817078 PMC10563303

[123] Liu F Korc M . Cdk4/6 inhibition induces epithelial-mesenchymal transition and enhanc es invasiveness in pancreatic cancer cells. Mol Cancer Ther. (2012) 11:2138–48. doi: 10.1158/1535-7163.MCT-12-0562. PMID: 22869556 PMC3752412

[124] Lee T Kim K Lee J Park SH Park YS Lim HY . Antitumor activity of sorafenib plus cdk4/6 inhibitor in pancreatic pa tient derived cell with kras mutation. J Cancer. (2018) 9:3394–9. doi: 10.7150/jca.26068. PMID: 30271501 PMC6160685

[125] Hidalgo M Garcia-Carbonero R Lim K-H Messersmith WA Garrido-Laguna I Borazanci E . A preclinical and phase ib study of palbociclib plus nab-paclitaxel in patients with metastatic adenocarcinoma of the pancreas. Cancer Res Commun. (2022) 2:1326–33. doi: 10.1158/2767-9764.CRC-22-0072. PMID: 36970055 PMC10035387

[126] Kumarasamy V Ruiz A Nambiar R Witkiewicz AK Knudsen ES . Chemotherapy impacts on the cellular response to cdk4/6 inhibition: di stinct mechanisms of interaction and efficacy in models of pancreatic cancer. Oncogene. (2020) 39:1831–45. doi: 10.1038/s41388-019-1102-1. PMID: 31745297 PMC7047578

[127] Salvador-Barbero B Álvarez-Fernández M Zapatero-Solana E El Bakkali A Menéndez MDC López-Casas PP . Cdk4/6 inhibitors impair recovery from cytotoxic chemotherapy in pancr eatic adenocarcinoma. Cancer Cell. (2020) 37:340–353.e6. doi: 10.1016/j.ccell.2020.01.007. PMID: 32109375

[128] Aslam R Richards CE Fay J Hudson L Workman J Lee CL . Synergistic effects of the combination of Alpelisib (Pi3k inhibitor) a nd Ribociclib (Cdk4/6 inhibitor) in preclinical colorectal cancer mode ls. Int J Mol Sci. (2024) 25:13264. doi: 10.3390/ijms252413264. PMID: 39769028 PMC11676898

[129] Lee CL Cremona M Farrelly A Workman JA Kennedy S Aslam R . Preclinical evaluation of the cdk4/6 inhibitor palbociclib in combinat ion with a pi3k or mek inhibitor in colorectal cancer. Cancer Biol Ther. (2023) 24:2223388. doi: 10.1080/15384047.2023.2223388. PMID: 37326340 PMC10281467

[130] Pek M Yatim SMJM Chen Y Li J Gong M Jiang X . Oncogenic kras-associated gene signature defines co-targeting of cdk4/ 6 and mek as a viable therapeutic strategy in colorectal cancer. Oncogene. (2017) 36:4975–86. doi: 10.1038/onc.2017.120. PMID: 28459468

[131] Sorokin AV Kanikarla Marie P Bitner L Syed M Woods M Manyam G . Targeting ras mutant colorectal cancer with dual inhibition of mek and cdk4/6. Cancer Res. (2022) 82:3335–44. doi: 10.1158/0008-5472.CAN-22-0198. PMID: 35913398 PMC9478530

[132] Chen SH Gong X Zhang Y Van Horn RD Yin T Huber L . Raf inhibitor ly3009120 sensitizes ras or braf mutant cancer to cdk4/6 inhibition by abemaciclib via superior inhibition of phospho-rb and s uppression of cyclin d1. Oncogene. (2018) 37:821–32. doi: 10.1038/onc.2017.384. PMID: 29059158

[133] Sorah JD Moore DT Reilley MJ Salem ME Triglianos T McRee AJ . Phase ii single-arm study of palbociclib and cetuximab in anti-egfr na ïve patients with kras/nras/braf wild-type, metastatic colorectal canc er. Oncologist. (2025) 30:oyaf305. doi: 10.1093/oncolo/oyaf305. PMID: 40990835 PMC12526856

[134] Isermann T Schneider KL Wegwitz F De Oliveira T Conradi L-C Volk V . Enhancement of colorectal cancer therapy through interruption of the h sf1-hsp90 axis by p53 activation or cell cycle inhibition. Cell Death Differ. (2025) 32:1734–49. doi: 10.1038/s41418-025-01502-x. PMID: 40204953 PMC12432187

[135] Zhang L Liu W Zheng Z Zhang Q He Y Gu J . Allosteric cdc37 inhibitor disrupts chaperone complex to block cdk4/6 maturation. Angew Chem Int Ed Engl. (2025) 64:e202413618. doi: 10.1002/anie.202413618. PMID: 39582167

[136] Menon M Elliott R Bowers L Balan N Rafiq R Costa-Cabral S . A novel tankyrase inhibitor, msc2504877, enhances the effects of clini cal cdk4/6 inhibitors. Sci Rep. (2019) 9:201. doi: 10.1038/s41598-018-36447-4. PMID: 30655555 PMC6336890

[137] Noh J-Y Lee IP Han NR Kim M Min YK Lee S-Y . Additive effect of cd73 inhibitor in colorectal cancer treatment with cdk4/6 inhibitor through regulation of pd-l1. Cell Mol Gastroenterol Hepatol. (2022) 14:769–88. doi: 10.1016/j.jcmgh.2022.07.005. PMID: 35843546 PMC9424593

[138] Zhang J Zhou L Zhao S Dicker DT El-Deiry WS . The cdk4/6 inhibitor palbociclib synergizes with irinotecan to promote colorectal cancer cell death under hypoxia. Cell Cycle (Georgetown Tex). (2017) 16:1193–200. doi: 10.1080/15384101.2017.1320005. PMID: 28486050 PMC5499912

[139] Wang D Wang X Zhang Y Yu L An J Wang X . The combination of il-2 nanoparticles and palbociclib enhances the ant i-tumor immune response for colon cancer therapy. Front Immunol. (2024) 15:1309509. doi: 10.3389/fimmu.2024.1309509. PMID: 38352877 PMC10861758

[140] Feng Z Qin M Jiang J Wang M Zhang T Li Y . Iterative design of a prodrug nanocarrier for cell cycle arrest, immun e modulation, and enhanced t cell infiltration for colon cancer therap y. Nano Lett. (2025) 25:2820–30. doi: 10.1021/acs.nanolett.4c06018. PMID: 39929737

[141] Wu T Chen Z To KKW Fang X Wang F Cheng B . Effect of abemaciclib (ly2835219) on enhancement of chemotherapeutic a gents in abcb1 and abcg2 overexpressing cells *in vitro* and *in vivo*. Biochem Pharmacol. (2017) 124:29–42. doi: 10.1016/j.bcp.2016.10.015. PMID: 27816545

[142] Tamura K Mukai H Naito Y Yonemori K Kodaira M Tanabe Y . Phase i study of palbociclib, a cyclin-dependent kinase 4/6 inhibitor, in Japanese patients. Cancer Sci. (2016) 107:755–63. doi: 10.1111/cas.12932. PMID: 26991823 PMC4968608

[143] Doi T Hewes B Kakizume T Tajima T Ishikawa N Yamada Y . Phase i study of single-agent ribociclib in Japanese patients with adv anced solid tumors. Cancer Sci. (2018) 109:193–8. doi: 10.1111/cas.13428. PMID: 29059492 PMC5765307

[144] Wang B Han J Elisseeff JH Demaria M . The senescence-associated secretory phenotype and its physiological an d pathological implications. Nat Rev Mol Cell Biol. (2024) 25:958–78. doi: 10.1038/s41580-024-00727-x. PMID: 38654098

[145] Coppé J-P Patil CK Rodier F Sun Y Muñoz DP Goldstein J . Senescence-associated secretory phenotypes reveal cell-nonautonomous f unctions of oncogenic ras and the p53 tumor suppressor. PLoS Biol. (2008) 6:2853–68. doi: 10.1371/journal.pbio.0060301. PMID: 19053174 PMC2592359

[146] Cao L Li K Li Q Tong Q Wang Y Huang L . The controversial role of senescence-associated secretory phenotype (sasp) in cancer therapy. Mol Cancer. (2025) 24:283. doi: 10.1186/s12943-025-02475-8. PMID: 41204284 PMC12595836

[147] Lau L Porciuncula A Yu A Iwakura Y David G . Uncoupling the senescence-associated secretory phenotype from cell cyc le exit via interleukin-1 inactivation unveils its protumorigenic role. Mol Cell Biol. (2019) 39:e00586–18. doi: 10.1128/MCB.00586-18. PMID: 30988157 PMC6549465

[148] Chibaya L Murphy KC DeMarco KD Gopalan S Liu H Parikh CN . Ezh2 inhibition remodels the inflammatory senescence-associated secret ory phenotype to potentiate pancreatic cancer immune surveillance. Nat Cancer. (2023) 4:872–92. doi: 10.1038/s43018-023-00553-8. PMID: 37142692 PMC10516132

[149] Klepacki H Sieklucka B Kalafut J Kowalczuk K Surazynski A Mojzych M . Mm-129 counteracts 5-fluorouracil-induced cellular senescence in colon cancer via sirt1/stat3 signaling pathway. Cells. (2025) 14:1498. doi: 10.3390/cells14191498. PMID: 41090726 PMC12524155

